# NAP1L2 drives mesenchymal stem cell senescence and suppresses osteogenic differentiation

**DOI:** 10.1111/acel.13551

**Published:** 2022-01-15

**Authors:** Meilin Hu, Liangyu Xing, Li Zhang, Fan Liu, Sheng Wang, Ying Xie, Jingjing Wang, Hongmei Jiang, Jing Guo, Xin Li, Jingya Wang, Lei Sui, Changyi Li, Dayong Liu, Zhiqiang Liu

**Affiliations:** ^1^ Tianjin Medical University School of Stomatology Tianjin Medical University Tianjin China; ^2^ The Province and Ministry Co‐Sponsored Collaborative Innovation Center for Medical Epigenetics Tianjin Key Laboratory of Cellular Homeostasis and Human Diseases Department of Physiology and Pathophysiology School of Basic Medical Science Tianjin Medical University Tianjin China

**Keywords:** bmsc, NAP1L2, osteogenesis, senescence, senile osteoporosis

## Abstract

Senescence of bone marrow mesenchymal stem cells (BMSCs) impairs stemness and osteogenic differentiation, but the key regulators for senescence and the related osteogenesis are not well defined. Herein, we screened the gene expression profiles of human BMSCs from young and old donors and identified that elevation of the nucleosome assembly protein 1‐like 2 (NAP1L2) expression was correlated with BMSC senescence and impaired osteogenesis. Elevated NAP1L2 expression was observed in replicative cell senescence and induced cell senescence in vitro, and in age‐related senescent human and mouse BMSCs in vivo, concomitant with significantly augmented chromatin accessibility detected by ATAC‐seq. Loss‐ and gain‐of‐functions of NAP1L2 affected activation of NF‐κB pathway, status of histone 3 lysine 14 acetylation (H3K14ac), and chromatin accessibility on osteogenic genes in BMSCs. Mechanistic studies revealed that NAP1L2, a histone chaperone, recruited SIRT1 to deacetylate H3K14ac on promoters of osteogenic genes such as *Runx2*, *Sp7*, and *Bglap* and suppressed the osteogenic differentiation of BMSCs. Importantly, molecular docking analysis showed a possible bond between NAP1L2 and an anti‐aging reagent, the nicotinamide mononucleotide (NMN), and indeed, administration of NMN alleviated senescent phenotypes of BMSCs. In vivo and clinical evidence from aging mice and patients with senile osteoporosis also confirmed that elevation of NAP1L2 expression was associated with suppressed osteoblastogenesis. Taken together, our findings suggest that NAP1L2 is a regulator of both BMSC cell senescence and osteogenic differentiation, and provide a new theoretical basis for aging‐related disease.

## Graphical abstract

Expression level of NAP1L2 determines BMSCs senescence via regulating NF‐κB signaling pathway. Suppressed NAP1L2 facilitates osteogenic genes transcription through activating H3K14ac modification on the promoter regions in young MSCs. High NAP1L2 reduces H3K14ac modification by recruiting SIRT1 to the promoter regions in old MSCs.

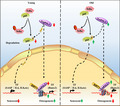

## 1 INTRODUCTION

Aging is a comprehensive process whose manifestations vary from cellular biological functions to gene transcription and protein post‐translational modifications (DiLoreto & Murphy, 2015; LeBrasseur et al., 2015). The causes of bone mass loss during aging are diverse, but it is generally agreed that age‐related bone loss is correlated with the imbalance of osteoblast‐mediated bone formation and osteoclasts‐mediated bone resorption in animals and in humans (Al‐Bari & Al Mamun, 2020). Stem cell exhaustion is considered one of the promoters of aging and contributes to the progressive decrease in tissue maintenance and repair (Liu et al., 2020; Wagner et al., 2019). Despite this knowledge, key regulators of aging and a detailed understanding of the biological mechanisms underpinning aging are elusive.

Bone marrow mesenchymal stem cells (BMSCs) have the potential for self‐renewal and serve as a lifelong reservoir for the generation of somatic cells and a powerful tool in bone tissue regenerative medicine (Sanghani‐Kerai et al., 2018; Uccelli et al., 2008). However, it has become apparent that the osteogenic differentiation of BMSCs decreases with senescence, which limits the potential of BMSCs in bone regeneration (Abdelmagid et al., 2015). Senescence of MSCs is a dynamic process accompanied by functional alterations in metabolism, genetic and epigenetic regulation of the transcriptome, and multiple signaling pathways (Mani et al., 2020; Wong et al., 2015). However, changes in these processes are probably a consequence of aging, and the real regulators governing BMSC senescence and osteogenesis are still unclear.

Nucleosome assembly protein 1 (NAP1) is a highly conserved gene family and is closely associated with nucleosome assembly, histone modifications, transcriptional regulation, and cell proliferation in various eukaryotes(Zlatanova et al., 2007). In humans and mice, the NAP1 gene family consists of at least five members, and previous studies have identified different roles of these molecules in regulating myocardial differentiation of induced pluripotent stem cells (iPSCs) (Gong et al., 2014), as well as homeostasis and hematopoietic differentiation of cord blood hematopoietic stem cells (Heshmati et al., 2018). Specifically, the *Nap1l2* gene plays an essential role in neural tube development and neural cell differentiation in mice (Rogner et al., 2000). It is also involved in the epigenetic regulation of gene expression during neuronal differentiation (Attia et al., 2013). Nevertheless, roles and mechanisms of NAP1L2 in regulating human BMSC senescence and osteogenesis have not yet been reported.

In the current study, we screened gene expression profiles of BMSCs derived from the young and the old donors, and identified *NAP1L2* gene as one of the most upregulated genes during aging, which expression was associated with impaired osteogenic differentiation of senescent BMSCs. We aim to clarify the role and mechanism of NAP1L2 in governing BMSC senescence and osteogenic differentiation through in vitro and in vivo experiments, as well as the correlation with senile osteoporosis in clinic.

## 2 RESULTS

### 2.1 BMSCs from elderly donors manifest senescent phenotypes and suppressed osteogenic differentiation capacity

Since the aim of this study is to identify key regulators governing BMSCs senescence and osteogenic differentiation, we firstly isolated BMSCs from the healthy young donors and the elderly donors, with the young donors aged between 17 and 45 years, and the elderly donors aged between 65 and 91 years. All these cells were examined by flow cytometry to ensure that CD90^+^CD44^+^CD19^−^CD45^−^ cells were purified for further experiments according to literature (Adesida et al., 2012) (Figure S1a). At the same time, osteogenic and adipogenic differentiation were conducted to guarantee the pluripotent stemness (Figure S1b). The positive rate of the β‐galactosidase staining was significantly increased in the old group compared with that in the young BMSCs (83 ± 1.56% vs. 28 ± 2.39%) (Figure S2a,b), and the expressions of the senescence‐related p21 and p16 were significantly elevated (Figure S2c,d). Moreover, the cell cycle of the old BMSCs was remarkably arrested at the G1 phase, indicating the proliferation capability was obviously suppressed (Figure S2e,f). Detection of telomere dysfunction‐induced foci (TIF) revealed that accumulation of telomere DNA damage, along with γ‐H2AX and 53BP1 was seen in the old BMSCs (Figure S2g,h). As to the osteogenic differentiation capacity, we assessed the alkaline phosphatase (ALP) activity and the mineralization of the extracellular matrix of BMSCs induced with osteogenic culture media for 7 or 14 days and confirmed that the old BMSCs had considerably impaired osteogenic differentiation capacity compared with the young BMSCs (Figure S3a–d). Meanwhile, the expressions of osteogenesis marker genes, such as *RUNX2*, *COL1A1*, and *ALP*, were all significantly decreased in the old BMSCs after induction (Figure S3e). Collectively, these data validated the stemness property of the BMSCs and confirmed the senescence phenotype of the old BMSCs.

### 2.2 Highly expressed NAP1L2 is correlated with BMSCs senescence

**FIGURE 1:**
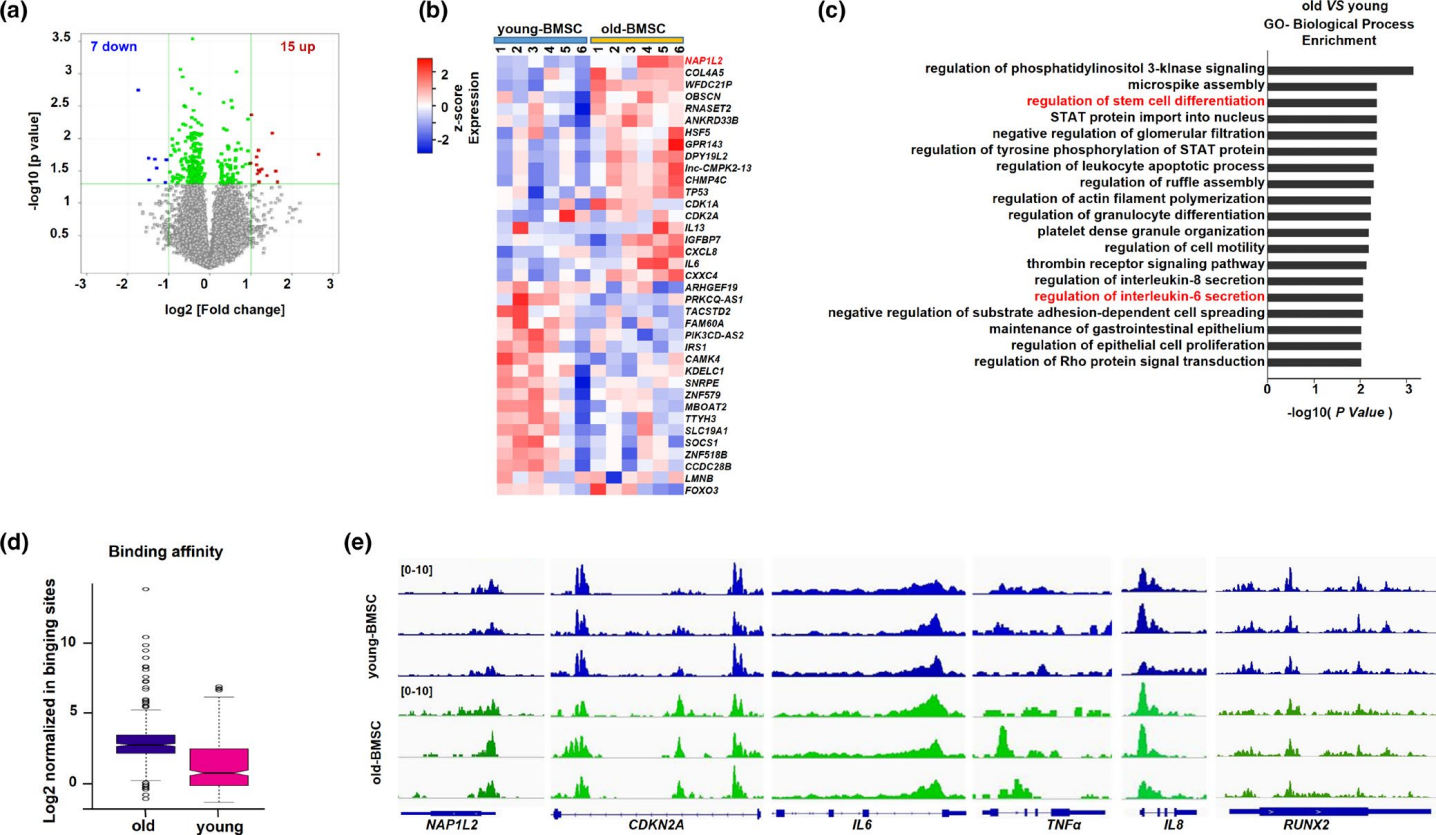
Highly expressed NAP1L2 is correlated with BMSC senescence. (a) Volcano plot showing differentially expressed genes in the young and the old BMSCs (old vs. young BMSCs, fold change ≥ 2, *p *≤ 0.05, *n* = 6). (b) Heat map of differently expressed genes (DEGs) by gene array (fold change ≥ 1.2, *p* ≤ 0.05). (c) GO analysis showing biological processes of the DEGs involved in the old BMSCs. (d) ATAC‐seq validating the global chromatin accessibility in the young (*n* = 3) and the old BMSCs (*n* = 3). (e) ATAC‐seq showing the chromatin accessibility of the *NAP1L2*, *CDKN2A*, *IL6*, *IL8*, *TNFα*, and *RUNX2* genes in the young (*n* = 3) and the old groups (*n* = 3)

Based on the corroboratory evidence for the BMSCs’ properties and senescence, we next performed gene array sequencing and the assay for transposase accessible chromatin with high‐throughput sequencing (ATAC‐seq) for six biologically independent samples from the old and the young BMSC groups (Figure S4a), respectively. Our screening analysis identified 15 upregulated and 7 downregulated genes in the old BMSCs compared with the young BMSCs (fold change ≥ 2, *p* ≤ 0.05) (Figure 1a), in which the expression level of the *NAP1L2* gene ranked at the top of all the differentially expressed genes (DEGs) in the old BMSCs, accompanied by high expression of senescence‐related secreted phenotype (SASP) factor genes (*TP53*, *IL6*, *and CCXL8*) (Figure 1b). Gene Ontology (GO) analysis showed that the biological processes involving these DEGs mainly involved stem cell differentiation and IL6 and IL8 secretion (Figure 1c). Kyoto Encyclopedia of Genes and Genomes (KEGG) analysis showed that the DEGs were primarily involved in the calcium signaling pathway and cAMP signaling pathway (Figure S4b). Compared with the young group, the global chromatin accessibility areas were significantly augmented in the old BMSCs (Figure 1d). We also observed that the chromatin accessibility of the *NAP1L2*, *IL6*, *IL8*, *CDKN2A*, and *TNFα* genes was remarkably increased, but that of the *RUNX2* gene was not obviously changed (Figure 1e). Simultaneously, using a replicative senescence BMSCs model, after the senescence phenotype was successfully validated by telomere dysfunction and DNA damage (Figure S5a), we further confirmed that NAP1L2 protein level was obviously upregulated (Figure S5b,c). Thus, our data suggest that the elevation of *NAP1L2* gene expression in the aging BMSCs was somehow correlated with chromatin modification and remodeling.

### 2.3 NAP1L2 expression is elevated in chemotherapeutic agents‐induced and oxidative stress‐induced cellular senescence models

To determine the biological function of NAP1L2 in senescence, we established the chemotherapeutic agents‐induced cellular senescence model with the mouse mesenchymal stem cell line C3H10T1/2 by etoposide as previously reported (Bang et al., 2019), and confirmed the senescent phenotype by β‐galactosidase staining (Figure S6a). Telomere FISH and immunostaining for γH2AX and 53BP1 revealed significantly increased telomere dysfunction‐induced foci in the etoposide treatment group (Figure S6b), under the condition that no visible apoptosis was induced by the reagent, since the cleaved caspase‐3 and PARP unchanged (Figure S6c). Quantification by qPCR confirmed that the expression of *p21* and SASP factors, such as *Il1α*, *Il1β*, *Il6*, and *Il8*, was successfully elevated along with our target gene *Nap1l2* (Figure S6d). Simultaneously, the protein levels of p53, p16, p21, and NAP1L2 were all upregulated with increasing dosage of etoposide treatment (Figure S6e). In addition, using an oxidative stress‐induced cell senescence model, whose senescent phenotype was validated by β‐galactosidase staining and telomere dysfunction assay without visible cell apoptosis (Figure S6f–i), we found that high expression of *Nap1l2*, *p21*, and SASP factors was successfully elicited by treatment of H_2_O_2_ (Figure S6j), and protein levels of NAP1L2, together with p53, p21, and p16 were all upregulated following H_2_O_2_ treatment (Figure S6k).

### 2.4 Manipulation of NAP1L2 alters cellular senescence progression through NF‐κB signaling pathway

**FIGURE 2:**
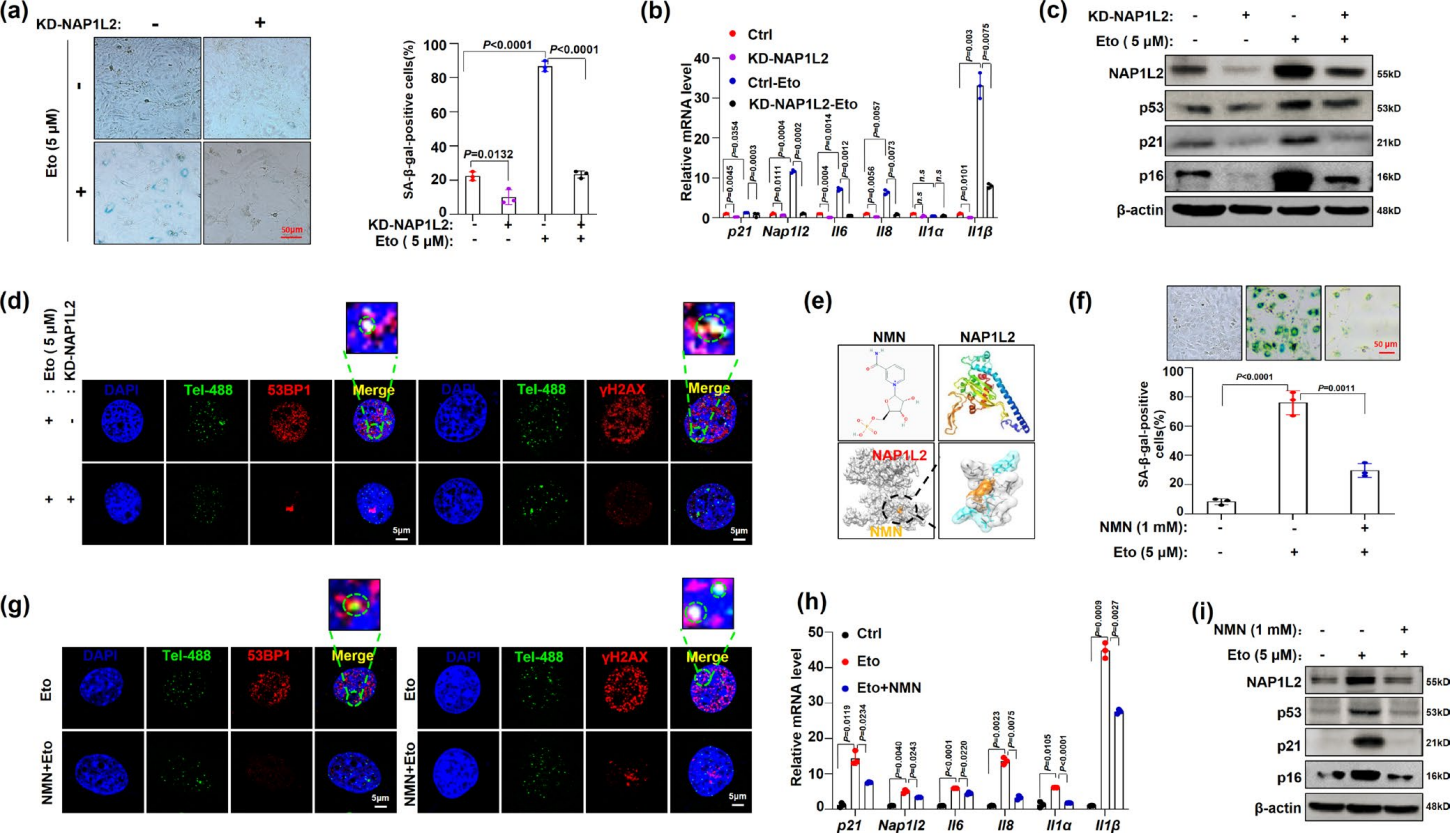
Manipulation of NAP1L2 expression alters cellular senescence progression. (a) β‐galactosidase staining and quantitative analysis of C3H10T1/2 cells with NAP1L2 knockdown and treated with 5 μM etoposide for 48 hr (*n* = 3). Scale bar, 50μm. (b) qPCR demonstrating the expression levels of *Nap1l2* and SASP factors of C3H10T1/2 with NAP1L2 knockdown and treated with 5 μM etoposide for 48 hr. (c) Protein levels of NAP1L2, p53, p21, and p16 in C3H10T1/2 cells with or without NAP1L2 knockdown and treatment with etoposide (5 μM) for 48 hr. (d) Representative images for 53BP1/γ‐H2AX at telomeres in C3H10T1/2 cells with or without NAP1L2 knockdown and treated with 5 μM etoposide for 48 hr. Scale bars, 5 μm. (e) Using the Swiss model server to predicted NAP1L2 protein structure and using Auto dock 4.0 to simulate anti‐aging drug (NMN) docking with NAP1L2. (f) β‐galactosidase staining and quantitative analysis of C3H10T1/2 cells treated with etoposide (5 μM) or NMN (1 mM) for 48 hr (*n* = 3). Scale bar, 50μm. (g) Representative images for 53BP1/γ‐H2AX at telomeres in C3H10T1/2 cells with treatment of etoposide (5 μM) or NMN (1 mM) for 48 hr (*n* = 3). Scale bars, 5 μm. (h) *Nap1l2* and SASP factors mRNA levels in C3H10T1/2 cells treated with etoposide (5 μM) or NMN (1 mM) for 48 hr (*n* = 3). (i) Representative western blots showing NAP1L2, p53, p21, and p16 protein levels in C3H10T1/2 cells treated with etoposide (5 μM) or NMN (1 mM) for 48 hr (*n* = 3). *p* values of mean ± SD were determined by Student's *t* test (*n* = 3)

**FIGURE 3:**
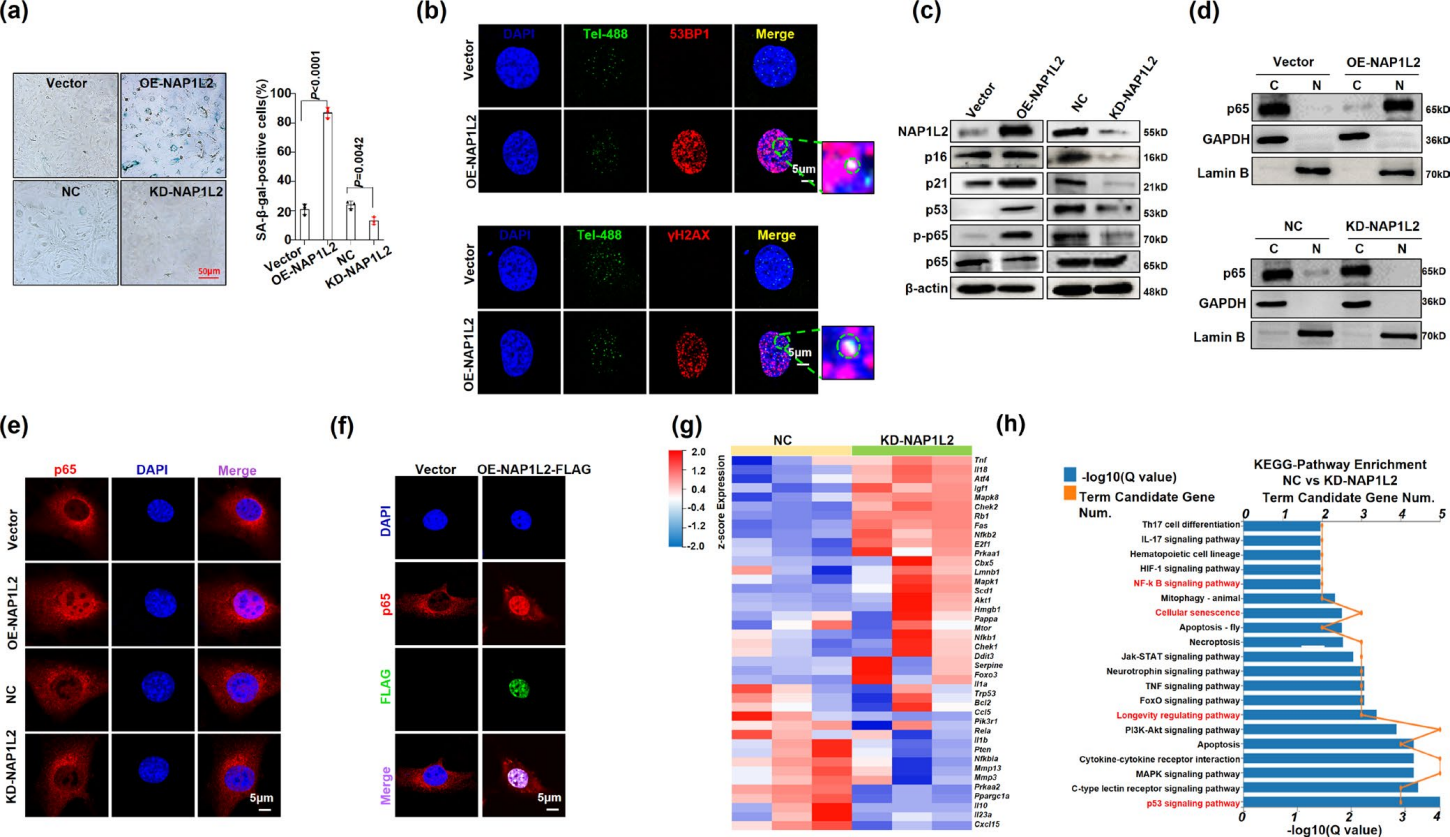
NAP1L2 regulates cellular senescence process through NF‐κB signaling pathway. (a) β‐galactosidase staining and quantitative analysis of positive C3H10T1/2 cells after overexpression or knockdown of NAP1L2 (*n* = 3). Scale bar, 50μm. (b) Representative images of 53BP1/γ‐H2AX at telomeres in C3H10T1/2 cells after overexpression of NAP1L2 (*n* = 3). Scale bars, 5 μm. (c) Western blot validating levels of p16, p21, p53, p‐p65, and p65 after NAP1L2 overexpression or knockdown. (d) Western blot to detect cytoplasm and nuclear p65 after NAP1L2 overexpression or knockdown. (e) Immunofluorescence showing subcellular localization of p65 after NAP1L2 overexpression or knockdown. Scale bar, 5μm. (f) Double immunostaining of p65 and FLAG in C3H10T1/2 cells after NAP1L2‐FLAG overexpression. Scale bar, 5μm. (g) Heat map showing the downregulation and upregulation senescence‐related genes in NAP1L2‐knockdown C3H10T1/2 cells. (h) KEGG Pathway analysis showing main signaling pathways of the DEGs involved in NAP1L2‐knockdown C3H10T1/2 cells. *p* values of mean ± SD were determined by Student's *t* test (*n* = 3)

Importantly, when we successfully manipulated the NAP1L2 expression with either lentivirus carrying shRNAs or vectors expressing mouse NAP1L2 in C3H10T1/2 cells (Figure S7a,b), we found that loss function of NAP1L2 significantly postponed the progression of etoposide‐induced senescence, evidenced by the suppression of positive rate of β‐gal staining and expression of the age‐related genes (Figure 2a–c), as well as reduction in telomere dysfunction‐induced foci in the senescent cells induced by etoposide (Figure 2d). Intriguingly, using a docking prediction tool, we found that small‐molecule aging antagonist nicotinamide mononucleotide (NMN) could dock with NAP1L2 protein (Figure 2e and Figure S7c,d); therefore, treatment with NMN was capable of alleviating the positive rate of the β‐galactosidase staining and telomere DNA damage in senescent cells (Figure 2f,g). NMN significantly suppressed the NAP1L2 protein level and expression of other SASP factor genes (Figure 2h,i and Figure S7e). On the contrary, overexpression of NAP1L2 increased the positive rate of β‐galactosidase staining and colocalizations of both 53BP1 and γ‐H2AX with telomere probe in the C3H10T1/2 cells (Figure 3a,b), and upregulated the expression of the SASP factor genes and *p21* concomitantly (Figure S7f). Since the NF‐κB signaling pathway is the prime signaling pathway governing aging in mammals (Osorio et al., 2012; Wang et al., 2019), we examined whether manipulation of NAP1L2 could affect the activation of the NF‐κB signaling pathway. Our results showed that overexpression of NAP1L2 augmented, but knockdown of NAP1L2 suppressed the p16, p21, p53, and phosphorylation level of p65 (Figure 3c). Moreover, the cytoplasm and nuclear fractions of p65 protein showed that majority of p65 was transported into the nucleus due to NAP1L2 overexpression, while knockdown of NAP1L2 hindered this transportation (Figure 3d). This phenomenon was also confirmed by immunofluorescence staining of cyto‐nuclear trans‐localization of p65 (Figure 3e), and p65 distinctly co‐localized with NAP1L2 after transported into the nucleus (Figure 3f).

Furthermore, we evaluated the alteration of the transcriptome due to NAP1L2 depletion, and the deferentially expressed genes in the heat map showed that levels of *Il1a*, *Il1b*, *Il10*, *Il23a (P19)*, *Cxcl15 (Il8)*, *Trp53*, *Bcl2*, and *Mmp3* were significantly decreased (Figure 3g). GO analysis of DEGs highlighted the involvement of the inflammatory response and immune response biological processes (Figure S7g), and KEGG pathway analysis showed that DEGs were mainly involved in the NF‐κB signaling pathway, cellular senescence, longevity regulating pathway, and p53 signaling pathway (Figure 3h). These findings suggest that NAP1L2 regulates cellular senescence through the NF‐κB signaling pathway and sequentially modulates the SASP factor expressions.

### 2.5 NAP1L2 is a key suppressor of osteogenic differentiation

**FIGURE 4:**
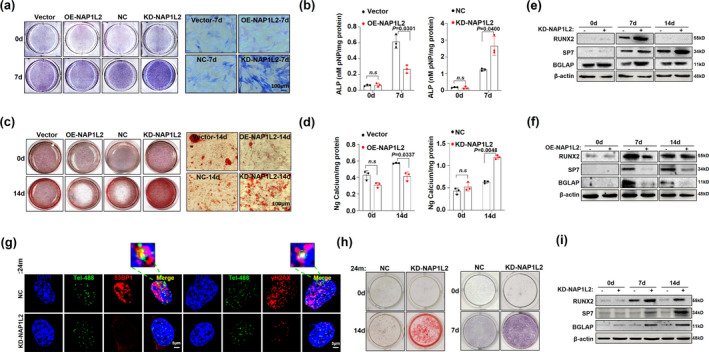
NAP1L2 is a key suppressor of osteogenic differentiation. (a) Alkaline phosphatase staining (ALP) and (b) quantitative analysis showing the osteogenic capability of C3H10T1/2 cells for osteogenesis induction 7 days after NAP1L2 overexpression or knockdown. (*n* = 3). Scale bar, 100μm. (c) Alizarin red staining and (d) calcium quantitative analysis to detect the mineralization ability of C3H10T1/2 cells after NAP1L2 overexpression or knockdown. (*n* = 3). Scale bar, 100μm. (e) Western blot showing protein levels of RUNX2, SP7, and BGLAP after NAP1L2 knockdown or (f) overexpression under the osteogenic induction. (g) Representative images of 53BP1/γ‐H2AX at telomeres after knockdown of NAP1L2 in BMSCs from senile mice. Scale bars, 5 μm. (h) Alkaline phosphatase staining (ALP) and Alizarin red staining demonstrating the osteogenic capability of 24‐month mice BMSCs after knocking down NAP1L2 under osteogenesis induction 7 days or 14 days. (i) Western blot showing the expression of RUNX2, SP7, and BGLAP in 24‐month mice BMSCs after knocking down NAP1L2 under osteogenesis induction 7 days or 14 days (*n* = 3). *p* values of mean ± SD were determined by Student's *t* test (*n* = 3)

To further clarify the relationship between NAP1L2 and osteogenesis, we then evaluated the effect of loss‐ and gain‐ of‐functions NAP1L2 on osteogenic differentiation in C3H10T1/2 cells. After induction with osteogenic media for 7 days, C3H10T1/2 cells with NAP1L2 knockdown showed an obvious increase in ALP staining and activity (Figure 4a,b), and they showed significant augmentation in mineralization of the extracellular matrix and calcium nodule accumulation at 14 days (Figure 4c,d), and *vice versa* when NAP1L2 was overexpressed. Similarly, the expression of osteogenesis‐related genes, such as *Runx2*, *Sp7*, *Bglap*, and *Spp1* was upregulated in the C3H10T1/2 cells due to loss function of NAP1L2, and suppressed in those with NAP1L2 overexpression at both mRNA and protein levels (Figure 4e,f and Figure S8a,b). Pharmacologically, administration of the anti‐aging reagent NMN also largely rescued the suppressive effect of NAP1L2 on osteogenic differentiation, evidenced by significantly restored Alizarin red staining and expression of osteogenic genes (Figure S8c–e).

We next validated the role of NAP1L2 in regulating BMSC senescence and osteogenesis in primary cells derived from young and aged mice. We observed that loss function of NAP1L2 significantly reduced the telomere dysfunction‐induced foci in BMSCs from senile mice (Figure 4g), and overexpression of NAP1L2 was capable of exacerbating telomere DNA damage in BMSCs from young mice (Figure S8f). Compared with the BMSCs from young mice, the expression of osteogenic genes in the BMSCs from aged mice was all significantly decreased along with augmentation of NAP1L2 expression, which was consistent with our previous in vitro results (Figure S8g). However, when NAP1L2 expression was interfered in the BMSCs from aged mice, the osteogenic differentiation capacity could be obviously rescued, evidenced by the significant increase in ALP activity and mineralization of the extracellular matrix (Figure 4h), as well as upregulated osteogenic genes after 7/14 days of induction (Figure 4i and Figure S8h). These data strongly suggest that NAP1L2 is an important suppressor for osteogenesis.

### 2.6 NAP1L2 recruits SIRT1 to deacetylate H3K14ac on promoters of osteogenic genes

**FIGURE 5:**
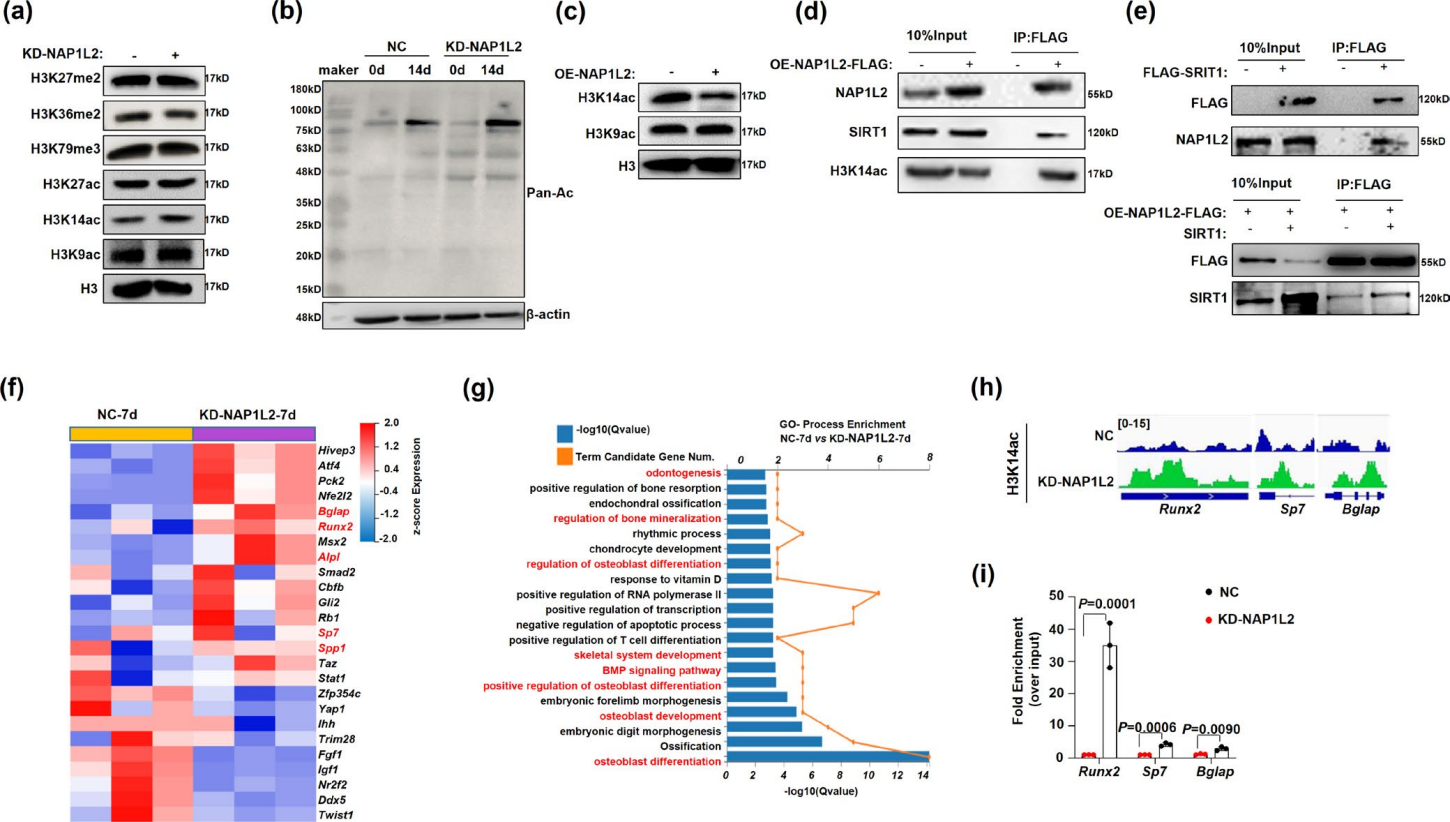
NAP1L2 recruits SIRT1 to deacetylate H3K14ac on promoters of osteogenic gene. (a) Global status of the most common histone modifications upon NAP1L2 depletion compared with the non‐target control. (b) Western blot validating the level of pan‐acetylation of the whole protein after knockdown of NAP1L2 under osteogenic induction. (c) Protein levels of H3K14ac and H3K9ac in C3H10T1/2 cells after NAP1L2 overexpression. (d) C3H10T1/2 cells infected with lentivirus carrying pITA‐insert‐mNap1l2‐FLAG plasmid for 72 h, co‐IP to detect the interaction between NAP1L2 and H3K14ac or SIRT1. (e) Co‐IP to show the interaction between NAP1L2 and SIRT1 in C3H10T1/2 cells infected with lentivirus carrying pCDH‐FLAG‐Sirt1, or infected together with lentivirus carrying pITA‐insert‐mNap1l2‐FLAG, and pcDNA6‐V5‐mSirt1. (f) Heat map showing the downregulation and upregulation osteogenic genes in NAP1L2‐knockdown C3H10T1/2 cells for osteogenic induction 7 days. (g) GO analysis showing biological processes of the DEGs involved in NAP1L2‐knockdown C3H10T1/2 cells under osteogenic induction. (h) Analysis of H3K14ac‐ChIP‐seq and (i) ChIP‐qPCR validating the recruitment of H3K14ac on the promoter regions of the osteogenic genes *Runx2*, *Sp7*, and *Bglap* in the Napl12 knockdown group (*n* = 3). *p* values of mean ± SD determined by Student's *t* test

Since a previous study revealed that NAP1L2 regulates neuronal differentiation through histone acetylation modification in an “on the gene” manner (Attia et al., 2007), we investigated whether NAP1L2 exerts its function through a similar epigenetic mechanism. We screened the global status of the most common histone modifications upon NAP1L2 depletion, including H3K27me2, H3K36me2, H3K79me3, H3K27ac, H3K14ac, and H3K9ac. We found only H3K14ac level was remarkably elevated compared with the non‐target control (Figure 5a). Meanwhile, pan‐acetylation of whole histones was greatly increased after osteogenic induction in the NAP1L2 depletion C3H10T1/2 cells (Figure 5b). On the contrary, when NAP1L2 was forcedly expressed, H3K14ac level was markedly inhibited, but H3K9ac level was not significantly affected (Figure 5c). Interestingly, after overexpression of NAP1L2, we found a direct interaction of NAP1L2 with H3K14ac and SIRT1 (Figure 5d,e). All of these findings suggested that NAP1L2 might reduce the level of H3K14 acetylation by interacting with SIRT1, thereby mediating chromatin remodeling and affecting osteogenesis. RNA sequencing results from C3H10T1/2 cells with NAP1L2 depletion and undergoing osteogenic induction for 7 days also revealed that osteogenic genes such as *Bglap*, *Runx2*, *Sp7*, and *Alp* were all increased significantly compared with the non‐target control group (Figure 5f). GO analysis for the DEGs further suggested participation in osteoblast differentiation, mineralization, osteoblastogenesis, BMP signaling, and skeleton development (Figure 5g). Moreover, the results of H3K14ac‐ChIP‐seq and ChIP‐qPCR showed that in the NAP1L2 knockdown group, acetylation modification of H3K14 on the promoter regions of the osteogenic genes *Runx2*, *Sp7*, and *Bglap* was increased significantly, which would benefit the expression of osteogenic genes and then promote osteogenic differentiation (Figure 5h,i). Thus, our present data elaborate a possible mechanism of NAP1L2 in regulating osteogenesis through H3K14 acetylation‐mediated chromosomal remodeling.

### 2.7 NAP1L2 expression is closely correlated with senile osteoporosis *in vivo* and in clinic

**FIGURE 6:**
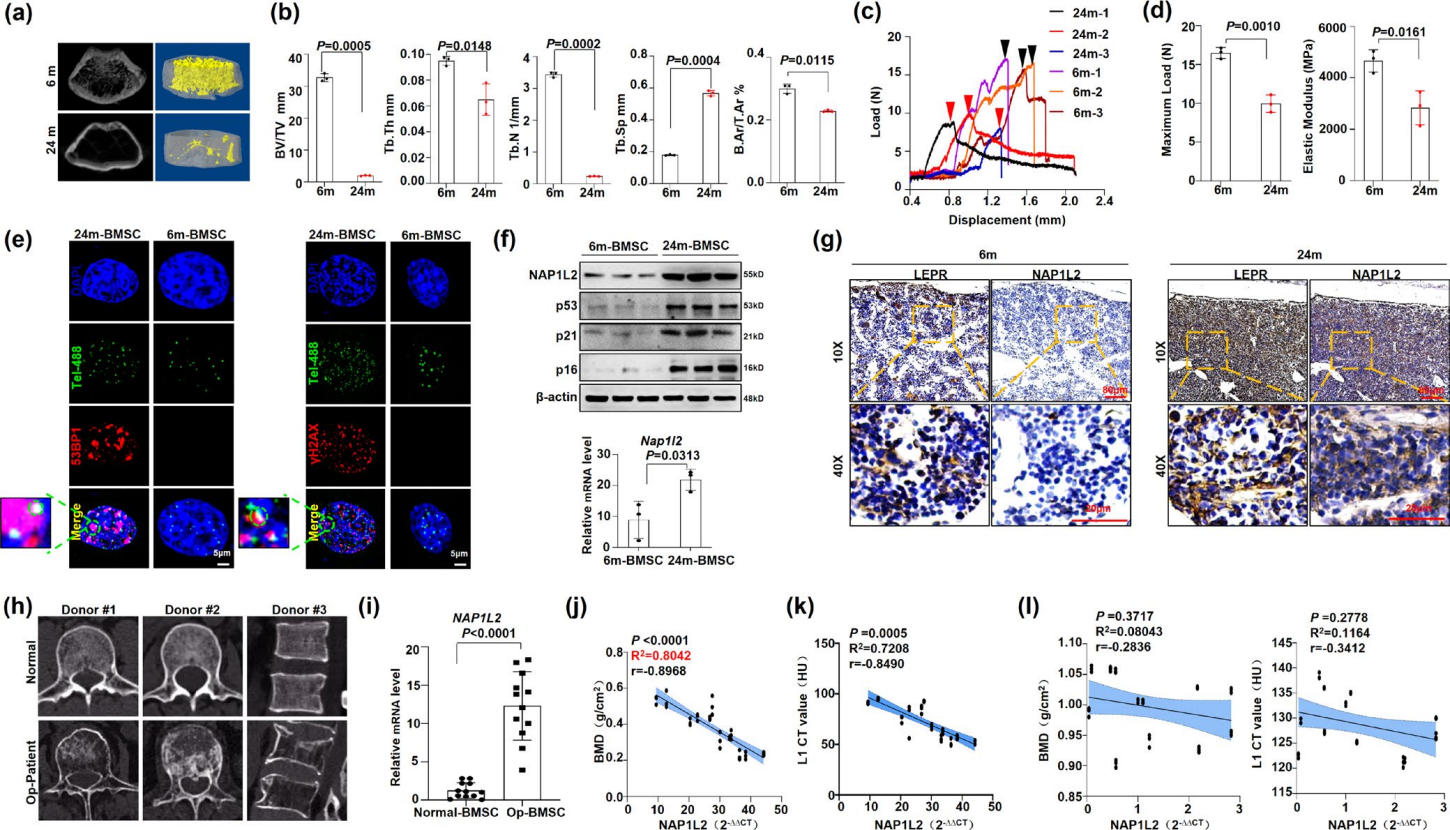
NAP1L2 is closely correlated with senile osteoporosis *in vivo* and in clinical. (a) Micro‐CT analysis to show bone mass and microstructure of senile osteoporosis mice. (b) Measurement of the percentage of trabecula separation (Tb.Sp), trabecular number (Tb.N), trabecular thickness (Tb.Th), bone volume/total volume (BV/TV), and percentage of cortical bone area to tissue area (B.Ar/T.Ar %) in the metaphyseal regions of femurs in the 6 months (*n* = 3) and 24 months mice (*n* = 3). (c) Three‐point bending test to detect the femur bone biomechanical strength of the young and old mice. The load‐displacement curves of the femurs in the 6‐month (*n* = 3) and 24‐month mice (*n* = 3). (d) The maximum load and elastic modulus of the femurs in the 6‐month (*n* = 3) and 24‐month mice (*n* = 3). (e) Representative images of 53BP1/γ‐H2AX at telomeres in 24‐month BMSCs (*n* = 3) and 6‐month BMSCs (*n* = 3). Scale bars, 5 μm. (f) Western blot and qPCR demonstrating NAP1L2, p53, p21, and p16 expression in 24‐month BMSCs (*n* = 3) and 6‐month BMSCs (*n* = 3). (g) Representative micrographs of immunohistochemistry staining for LEPR and NAP1L2 in femurs of the 6‐month (*n* = 3) and 24‐month mice (*n* = 3). (h) CT images of age‐paired healthy donors (*n* = 3) and patients with senile osteoporosis (*n* = 3). (i) qPCR showing *NAP1L2* expression in osteoporosis patients’ BMSCs (*n* = 12). (j) Correlation analysis of NAP1L2 and bone mineral density (BMD) in osteoporosis patients (*n* = 12). (k) Correlation analysis of NAP1L2 and CT value in osteoporosis patients (*n* = 12). (l) Correlation analysis of NAP1L2 and bone mineral density (BMD) or CT value in age‐paired healthy donors (*n* = 12). *p* values of mean ± SD determined by Student's *t* test

To verify the important role of NAP1L2 *in vivo*, we successfully established a senile osteoporosis model in 24‐month‐old mice and confirmed obvious bone marrow loss in the aged mice using a micro‐CT scan by detecting the microstructure of the femur (Figure 6a), as well as trabecular number (Tb.N), trabecular thickness (Tb. Th), bone volume/total volume (BV/TV), trabecular separation (Tb. Sp), and percentage of cortical bone area to tissue area (B. Ar/T. Ar %) (Figure 6b). At the same time, three‐point bending test evaluating the biomechanical femur bone strength of the young and senile mice showed that the maximum load and elastic modulus of senile mice were significantly reduced compared with the young mice (Figure 6c,d). When the BMSCs were isolated from the young and aged mice, the telomere dysfunction assay showed significantly increased colocalizations of both 53BP1 and γ‐H2AX with telomere probe in BMSCs from the aged mice (Figure 6e), along with the markedly elevated NAP1L2 expression both at mRNA and protein levels (Figure 6f). Immunohistochemistry staining for NAP1L2 level in femur sections of osteoporotic mice showed that the expression was significantly higher in the leptin receptor positive (LEPR^+^) BMSCs than that in the control group (Figure 6g). Notably, when we isolated BMSCs from patients with osteoporosis (Figure 6h), we also observed significantly higher *NAP1L2* expression than those from age‐paired healthy controls (Figure 6i). A significant negative correlation was elicited between *NAP1L2* expression and bone mineral density (BMD), as well as the CT value for the L1 lumbar in osteoporosis patients (Figure 6j,k); however, no significant correlation between *NAP1L2* expression and bone mineral density (BMD) or CT value in the age‐paired healthy donors was observed (Figure 6l). Collectively, these *in vivo* and clinical data strongly demonstrate a close relationship between senile osteoporosis and NAP1L2 expression.

## 3 DISCUSSION

Aging is such a complicated process that the key drivers and regulators are not easy to define. In the current study, we report that NAP1L2, whose expression is significantly elevated during aging, has a close correlation with physiological and pathophysiological senescence as well as with the suppressed osteogenic capabilities of BMSCs. We also revealed a possible regulatory mechanism of NAP1L2 in activating the NF‐κB pathway and impairing osteogenic potential through epigenetic regulation of histone acetylation at H3K14.

Ample studies have revealed that aging leads to diverse changes in cellular biology and the transcriptome regulated by genomic or epigenetic mechanisms (Declerck & Vanden Berghe, 2018; Field et al., 2018). In our experiment, we firstly confirmed that BMSCs derived from elderly donors showed a significant senescent phenotype compared with those from young donors, evidenced by increasing β‐galactosidase staining, upregulated age‐related genes *p21* and *p16*, cell cycle arrest at the G1 phase, limited proliferation, augmented telomere DNA damage, and suppressed osteogenic differentiation capacity. Based on these findings, we performed gene array sequencing to screen differentially expressed genes and identified 21 differently expressed genes between the two groups, with 15 being elevated and 7 being suppressed. Our focus on the *Nap1l2* gene is based upon Rogner's series of work showing that NAP1L2 plays a critical role in neuronal differentiation in mice (Attia et al., 2007, 2013; Rogner et al., 2000). In our study, we also observed that the elevated NAP1L2 in the old BMSCs was concomitant with the augmentation of chromatin accessibility, which was in line with the previous finding that NAP1L2 is an epigenetic regulator of nucleosome assembly via histone acetylation modification (Attia et al., 2007). Moreover, our GO analysis showed that IL6 and IL8 secretion is one of the most changed biological processes, along with stem cell differentiation. In fact, IL6 and IL8 are important components of senescence‐associated secretory phenotype (SASP) (Gorgoulis et al., 2019). In our *in vitro*‐induced cellular senescence model and in BMSCs from the aged mice, depletion of NAP1L2 significantly alleviated the process of cellular senescence. Concomitantly, SASP factors, such as IL6 and IL8 secretion, activation of the NF‐κB signaling pathway, and activation of the p53 signaling pathway were all remarkably diminished. In sum, our data suggest that NAP1L2 regulates the cellular senescence process through the NF‐κB signaling pathway and thus identifies NAP1L2 as an important regulator of the senescence of BMSCs.

It has become apparent that the osteogenic differentiation of BMSCs decreases with senescence, which limits the potential of BMSCs in bone regeneration (Abdelmagid et al., 2015; D'Ippolito et al., 1999). Our study also evaluated the impact of NAP1L2 on the osteogenesis of BMSCs, and the results indicated that deletion of Nap1l2 resulted in a significant amelioration of the osteogenic differentiation of BMSCs. Mechanistically, NAP1L2 likely participates in restraining H3K14 acetylation (H3K14ac) on promoters of osteogenic genes, such as *Bglap*, *Runx2*, and *Sp7*, because depletion of NAP1L2 significantly augmented the global H3K14ac and expression of these genes. These findings were slightly different from those of a previous study, which reported that NAP1L2 coexists with chromatin in the nucleus and facilitates histone acetyltransferase activity to histones H3 and H4 (Attia et al., 2007). Interestingly, our results also suggest that NAP1L2 actually recruits the NAD^+^‐dependent deacetylase sirtuin‐1 (SIRT1), and the latter reduces the level of H3K14ac on promoters of osteogenic genes, since H3K14ac is generally linked to gene activation (Li et al., 2016). Thus, we uncovered a previously unreported role of NAP1L2 in regulating osteogenic differentiation. However, we are not sure whether NAP1L2 only affects cell senescence and osteogenic differentiation at the same time or inhibits osteogenic differentiation by inducing cell senescence.

Finally, using an *in vivo* aging mouse model and clinical sample from patients with senile osteoporosis, we further validated the correlation of elevated NAP1L2 expression with the development of aging and impairment of BMSCs. Similar to our above results, deletion of NAP1L2 improved the osteogenesis of the senescent BMSCs. Therefore, we verified that augmentation of NAP1L2 expression is a barrier to bone formation. Translationally, our study also suggests that nicotinamide mononucleotide (NMN), a crucial NAD + intermediate that decreases with age in mammals (Song et al., 2019), binds NAP1L2 protein, and suppresses its expression. Other studies have verified that supplementation of NMN has preventive effects against age‐associated pathophysiology and disease conditions (Yoshino et al., 2018), and also clarifies that administration of NMN reduces senescent phenotypes of BMSCs and ameliorates osteogenic differentiation.

Hence, our findings suggest that NAP1L2 serves as a new theoretical basis for aging‐related disease. The translational merit of our study is to indicate that the aging antagonist reagent nicotinamide mononucleotide (NMN) has an inhibitory effect on NAP1L2 expression, thus providing a theoretical basis for the management of age‐related diseases.

## 4 EXPERIMENTAL PROCEDURES

### 4.1 Animals and treatment procedure

We divided the mice into two groups: the old C57 mice (24 months old) and the young C57 mice (6 months old). Mice were sacrificed under 10% chloral hydrate anesthesia. Both sides of the femur were collected and fixed in 4% paraformaldehyde. The femur samples were first dehydrated using 70% ethanol for 48 h. Samples were scanned using a micro‐CT scanner (Bruker microCT, Kontich, Belgium). The ROI is superiorly placed 1.0 mm from the distal growth plate of the femoral head within the femoral cortex. All images were post‐operated to isolate cancellous bone from cortical bone and preserve its morphology using a threshold of 800 manually. Scans were repeated three times, bone micro‐architectural parameters of each group were taken from the same region of interest (ROI). The following structural parameters of the ROI were calculated: bone volume/tissue volume (BV/TV), trabecular number (Tb.N), trabecular thickness (Tb.Th), trabecular separation (Tb.Sp), and percentage of cortical bone area to total tissue area (B.Ar/T.Ar %).

### 4.2 Cell culture

The samples were divided into groups according to age, the young group (young BMSC, 17–45 years old, *n* = 6), the old group (old‐BMSC, 65–91 years old, *n* = 6). BMSCs were taken from patients undergoing fracture clean‐up and requiring bone marrow examination. Exclusion criteria include malignant tumors, family genetic diseases, and other bone diseases. All patients have signed informed consent.

Density gradient centrifugation was used to isolate adult bone marrow mononuclear cells (MNC), and BMSCs were purified by the characteristics of differential attachment. BMCSs were plated with Dulbecco's modified Eagle's medium (DMEM) including 15% fetal bovine serum (FBS) (Gibco, Life Technologies, USA), 100 U/ml penicillin, 100 μg/ml streptomycin. The medium was replaced every 3 days. After the cell fusion degree reaches 80–90%, the cells are passaged, and passages 3 can be used for subsequent experiments. BMCSs were passaged 1:2 when cultures reached confluency. Cultures were defined as replicative senescent BMCSs when they failed to reach confluency 10 d after a 1:2 split. Primary mouse BMSCs were isolated from C57BL/6J mice at 6 or 24 months by flushing the bone marrow of femurs. Cells were cultured in minimum essential medium α (α‐MEM) supplemented with 15% FBS. Human embryonic kidney 293 T cells and multipotent mouse mesenchymal stem cells (C3H10T1/2) were maintained in DMEM medium with 10% FBS.

### 4.3 Plasmid construction and viral infection

Plasmids expressing shRNAs targeting Nap1l2 were purchased from Genechem (Shanghai, China). For NAP1L2 overexpression, Nap1l2 cDNA was cloned into the pITA‐insert vector. Transient transfections to HEK293T cells were performed using polyethyleneimine (PEI) (Polysciences, USA) in the OPTI‐MEM medium (Life Technologies, Carlsbad, CA, USA) with a ratio of 1:4 of DNA: PEI. Viral particles were produced by HEK293T cells transfected with 4 μg PMD2G and 6 μg PSPAX2 packaging plasmids (Addgene, USA), together with 8 μg lentiviral expressing vectors encoding target genes (pITA‐insert‐mNap1l2‐FLAG, shNap1l2, pCDH‐FLAG‐Sirt1, pcDNA6‐V5‐mSirt1). Supernatant carrying the viral particles was harvested after transfection and concentrated to 1/100 volume by poly (ethylene glycol) 8,000 (Sigma‐Aldrich, USA). For viral infection, 1 × 10^6^ C3H10T1/2 cells were seeded and then added 20 μl viral concentration and 8 μg/ml polybrene, and cells were spun at 200 ×g for 45 min at 20°C. 12 h after infection, the medium was changed and cells were cultured for another 48 h until further management. Western blots and qPCR assessed efficiencies of knockdown and overexpression. Plasmids of pITA‐insert‐mNap1l2‐FLAG, pCDH‐FLAG‐Sirt1, and pcDNA6‐V5‐mSirt1 were constructed by our team.

### 4.4 Alizarin red Staining and calcium quantitative analysis

After cultured with osteoblast induction medium for 14 days, the cells were washed twice with PBS and fixed at room temperature for 15 min with 95% ethanol and washed with deionized water. The fixed cells were incubated with 40 mM (PH: 4.2) Alizarin red staining solution (Sigma‐Aldrich, USA) for 10 min at room temperature and then washed thoroughly in deionized water. To quantitate the calcium mineral content, the stained cells were dissolved with 10% cetylpyridinium chloride for 60 min at room temperature. Concentrations were determined by reading the absorbance at 562 nm using a standard calcium curve. The final calcium level in each group was normalized to the total protein concentrations obtained from a duplicate plate.

### 4.5 Immunofluorescence‐telomere FISH (IF‐FISH) of telomere dysfunction‐induced foci assay

Fluorescence in situ hybridization (FISH) of telomeres was performed on 2% formaldehyde fixed cells using a PNA‐FISH with a (CCCTAA)3‐Alexa488 telomere probe (Panagene, Daejeon, KR). 53BP1/γ‐H2AX immunofluorescence‐telomere FISH was carried out as described by Kasbek and colleagues(Kasbek et al., 2013). The primary antibody and the Alexa Fluor^TM^ 555‐conjugated secondary antibody were both diluted 1:500. Stained cells were photographed using an Olympus inverted microscope or confocal microscope (Olympus FV1000, JP). All antibodies, vendors were provided in the key resources table.

### 4.6 Co‐immunoprecipitation and Chromatin‐Immunoprecipitation (ChIP)

Co‐IP was conducted according to a procedure previously described (Liu et al., 2014). For the ChIP assay, 1 × 10^6^ cells were crosslinked in 1% formaldehyde (Thermo Scientific, USA) for 10 min. Crosslinking was quenched with glycine, and cells were washed two times with ice PBS. The ChIP of H3K14ac (Abcam, UK) cells was lysed in lysis‐buffer and chromatin was sonicated to a fragment size of 150 to 300 bp by using eight cycles. The chromatin concentration and chromatin fragments were checked using Nanodrop and 2% agarose gel. Then, samples were immunoprecipitated using an anti‐H3K14ac antibody linked to magnetic beads and the DNA purified with QIAquick PCR Purification Kit (QIAGEN, USA). The normalized enrichment value was calculated as the subtraction of the IP relative value with the input relative value.

### 4.7 The assay for transposase accessible chromatin with high‐throughput sequencing (ATAC‐seq)

ATAC‐seq was performed as previously described (Buenrostro et al., 2015). 5 × 10^3^–5 × 10^4^ cells were used for each tagmentation using Tn5 transposases. The resulting DNA was isolated, quantified, and sequenced on an Illumina NextSeq500 system. The raw reads were aligned to the human genome assembly hg19 using Bowtie40 with the default parameters, and only tags that uniquely mapped to the genome were used for further analysis.

### 4.8 Statistical analysis

All quantitative data are shown as mean ± SD for at least three independent experiments. Differences between groups were determined using paired two‐tailed sided Student's *t* test or two‐way ANOVA. Pearson correlation test was used to determine the correlations between gene expressions. A *p* value < 0.05 was considered statistically significant compared with the controls, respectively.

## CONFLICT OF INTEREST

All authors declare no conflict of interest.

## AUTHOR CONTRIBUTIONS

Z.Q.L., D.Y.L., and C.Y.L. contributed to the design of the experiments. M.L.H, L.Y.X, J.Y.W, J.J.W., and L.Z. contributed to writing the manuscript. M.L.H, L.Y.X, Y.X., F.L., H.M.J, J.G., L.S., and X.L. contributed to performing the experiments and statistical analyses. M.L.H., L.Z., and S.W. were in charge of the animal studies. M.L.H and L.Y.X provided the clinical statistics. D.Y. L. and Z.Q.L. approved the final submission.

## Supporting information

Supplementary Material

Fig S1‐S8

Method S1

## Data Availability

All data needed to evaluate the conclusions in the paper are present in the paper and/or the supplementary files, and the RNA‐seq and ChIP‐seq data can be publicly found at the Gene Expression Omnibus database under accession number GSE166244. Requests for any materials in this study should be directed to Zhiqiang Liu and obtained through an MTA.

## References

[acel13551-bib-0001] Abdelmagid, S. M. , Barbe, M. F. , & Safadi, F. F. (2015). Role of inflammation in the aging bones. Life Sciences, 123, 25–34. 10.1016/j.lfs.2014.11.011 25510309

[acel13551-bib-0002] Adesida, A. B. , Mulet‐Sierra, A. , & Jomha, N. M. (2012). Hypoxia mediated isolation and expansion enhances the chondrogenic capacity of bone marrow mesenchymal stromal cells. Stem Cell Research & Therapy, 3(2), 9. 10.1186/scrt100 22385573 PMC3392769

[acel13551-bib-0003] Al‐Bari, A. A. , & Al Mamun, A. (2020). Current advances in regulation of bone homeostasis. FASEB BioAdvances, 2(11), 668–679. 10.1096/fba.2020-00058 33205007 PMC7655096

[acel13551-bib-0004] Attia, M. , Rachez, C. , Avner, P. , & Rogner, U. C. (2013). Nucleosome assembly proteins and their interacting proteins in neuronal differentiation. Archives of Biochemistry and Biophysics, 534(1–2), 20–26. 10.1016/j.abb.2012.09.011 23031499

[acel13551-bib-0005] Attia, M. , Rachez, C. , De Pauw, A. , Avner, P. , & Rogner, U. C. (2007). Nap1l2 promotes histone acetylation activity during neuronal differentiation. Molecular and Cellular Biology, 27(17), 6093–6102. 10.1128/MCB.00789-07 17591696 PMC1952155

[acel13551-bib-0006] Bang, M. , Kim, D. G. , Gonzales, E. L. , Kwon, K. J. , & Shin, C. Y. (2019). Etoposide induces mitochondrial dysfunction and cellular senescence in primary cultured rat astrocytes. Biomolecules & Therapeutics, 27(6), 530–539. 10.4062/biomolther.2019.151 31646843 PMC6824621

[acel13551-bib-0007] Buenrostro, J. D. , Wu, B. , Chang, H. Y. , & Greenleaf, W. J. (2015). ATAC‐seq: A method for assaying chromatin accessibility genome‐wide. Current Protocols in Molecular Biology, 109(1), 21 29 21–21 29 29. 10.1002/0471142727.mb2129s109 PMC4374986 25559105

[acel13551-bib-0008] Declerck, K. , & Vanden Berghe, W. (2018). Back to the future: Epigenetic clock plasticity towards healthy aging. Mechanisms of Ageing and Development, 174, 18–29. 10.1016/j.mad.2018.01.002 29337038

[acel13551-bib-0009] DiLoreto, R. , & Murphy, C. T. (2015). The cell biology of aging. Molecular Biology of the Cell, 26(25), 4524–4531. 10.1091/mbc.E14-06-1084 26668170 PMC4678010

[acel13551-bib-0010] D'Ippolito, G. , Schiller, P. C. , Ricordi, C. , Roos, B. A. , & Howard, G. A. (1999). Age‐related osteogenic potential of mesenchymal stromal stem cells from human vertebral bone marrow. Journal of Bone and Mineral Research, 14(7), 1115–1122. 10.1359/jbmr.1999.14.7.1115 10404011

[acel13551-bib-0011] Field, A. E. , Robertson, N. A. , Wang, T. , Havas, A. , Ideker, T. , & Adams, P. D. (2018). DNA methylation clocks in aging: Categories, causes, and consequences. Molecular Cell, 71(6), 882–895. 10.1016/j.molcel.2018.08.008 30241605 PMC6520108

[acel13551-bib-0012] Gong, H. , Yan, Y. , Fang, B. O. , Xue, Y. , Yin, P. , Li, L. U. , Zhang, G. , Sun, X. , Chen, Z. , Ma, H. , Yang, C. , Ding, Y. , Yong, Y. E. , Zhu, Y. , Yang, H. , Komuro, I. , Ge, J. , & Zou, Y. (2014). Knockdown of nucleosome assembly protein 1‐like 1 induces mesoderm formation and cardiomyogenesis via notch signaling in murine‐induced pluripotent stem cells. Stem Cells, 32(7), 1759–1773. 10.1002/stem.1702 24648372

[acel13551-bib-0013] Gorgoulis, V. , Adams, P. D. , Alimonti, A. , Bennett, D. C. , Bischof, O. , Bishop, C. , Campisi, J. , Collado, M. , Evangelou, K. , Ferbeyre, G. , Gil, J. , Hara, E. , Krizhanovsky, V. , Jurk, D. , Maier, A. B. , Narita, M. , Niedernhofer, L. , Passos, J. F. , Robbins, P. D. , … Demaria, M. (2019). Cellular senescence: Defining a path forward. Cell, 179(4), 813–827. 10.1016/j.cell.2019.10.005 31675495

[acel13551-bib-0014] Heshmati, Y. , Kharazi, S. , Türköz, G. , Chang, D. , Kamali Dolatabadi, E. , Boström, J. , Krstic, A. , Boukoura, T. , Wagner, E. , Kadri, N. , Månsson, R. , Altun, M. , Qian, H. , & Walfridsson, J. (2018). The histone chaperone NAP1L3 is required for haematopoietic stem cell maintenance and differentiation. Scientific Reports, 8(1), 11202. 10.1038/s41598-018-29518-z 30046127 PMC6060140

[acel13551-bib-0015] Kasbek, C. , Wang, F. , & Price, C. M. (2013). Human TEN1 maintains telomere integrity and functions in genome‐wide replication restart. Journal of Biological Chemistry, 288(42), 30139–30150. 10.1074/jbc.M113.493478 PMC3798482 24025336

[acel13551-bib-0016] LeBrasseur, N. K. , Tchkonia, T. , & Kirkland, J. L. (2015). Cellular senescence and the biology of aging, disease, and frailty. Nestlé Nutrition Institute Workshop Series, 83, 11–18. 10.1159/000382054 26485647 PMC4780350

[acel13551-bib-0017] Li, N. A. , Li, Y. , Lv, J. , Zheng, X. , Wen, H. , Shen, H. , Zhu, G. , Chen, T.‐Y. , Dhar, S. S. , Kan, P.‐Y. , Wang, Z. , Shiekhattar, R. , Shi, X. , Lan, F. , Chen, K. , Li, W. , Li, H. , & Lee, M. G. (2016). ZMYND8 reads the dual histone mark H3K4me1‐H3K14ac to antagonize the expression of metastasis‐linked genes. Molecular Cell, 63(3), 470–484. 10.1016/j.molcel.2016.06.035 27477906 PMC4975651

[acel13551-bib-0018] Liu, J. , Ding, Y. , Liu, Z. , & Liang, X. (2020). Senescence in mesenchymal stem cells: functional alterations, molecular mechanisms, and rejuvenation strategies. Frontiers in Cell and Developmental Biology, 8, 258. 10.3389/fcell.2020.00258 32478063 PMC7232554

[acel13551-bib-0019] Liu, Z. , Li, T. , Reinhold, M. I. , & Naski, M. C. (2014). MEK1‐RSK2 contributes to Hedgehog signaling by stabilizing GLI2 transcription factor and inhibiting ubiquitination. Oncogene, 33(1), 65–73. 10.1038/onc.2012.544 23208494

[acel13551-bib-0020] Mani, C. , Reddy, P. H. , & Palle, K. (2020). DNA repair fidelity in stem cell maintenance, health, and disease. Biochimica Et Biophysica Acta (BBA) ‐ Molecular Basis of Disease, 1866(4), 165444. 10.1016/j.bbadis.2019.03.017 PMC6935429 30953688

[acel13551-bib-0021] Osorio, F. G. , Lopez‐Otin, C. , & Freije, J. M. (2012). NF‐kB in premature aging. Aging (Albany NY), 4(11), 726–727. 10.18632/aging.100502 23211391 PMC3560436

[acel13551-bib-0022] Rogner, U. C. , Spyropoulos, D. D. , Le Novere, N. , Changeux, J. P. , & Avner, P. (2000). Control of neurulation by the nucleosome assembly protein‐1‐like 2. Nature Genetics, 25(4), 431–435. 10.1038/78124 10932189

[acel13551-bib-0023] Sanghani‐Kerai, A. , McCreary, D. , Lancashire, H. , Osagie, L. , Coathup, M. , & Blunn, G. (2018). Stem cell interventions for bone healing: Fractures and osteoporosis. Current Stem Cell Research & Therapy, 13(5), 369–377. 10.2174/1574888X13666180410160511 29637866

[acel13551-bib-0024] Song, J. , Li, J. , Yang, F. , Ning, G. , Zhen, L. , Wu, L. , Zheng, Y. , Zhang, Q. I. , Lin, D. , Xie, C. , & Peng, L. (2019). Nicotinamide mononucleotide promotes osteogenesis and reduces adipogenesis by regulating mesenchymal stromal cells via the SIRT1 pathway in aged bone marrow. Cell Death & Disease, 10(5), 336. 10.1038/s41419-019-1569-2 31000692 PMC6472410

[acel13551-bib-0025] Uccelli, A. , Moretta, L. , & Pistoia, V. (2008). Mesenchymal stem cells in health and disease. Nature Reviews Immunology, 8(9), 726–736. 10.1038/nri2395 19172693

[acel13551-bib-0026] Wagner, D. R. , Karnik, S. , Gunderson, Z. J. , Nielsen, J. J. , Fennimore, A. , Promer, H. J. , Lowery, J. W. , Loghmani, M. T. , Low, P. S. , McKinley, T. O. , Kacena, M. A. , Clauss, M. , & Li, J. (2019). Dysfunctional stem and progenitor cells impair fracture healing with age. World J Stem Cells, 11(6), 281–296. 10.4252/wjsc.v11.i6.281 31293713 PMC6600851

[acel13551-bib-0027] Wang, Y. , Wang, L. , Wen, X. , Hao, D. , Zhang, N. , He, G. , & Jiang, X. (2019). NF‐kappaB signaling in skin aging. Mechanisms of Ageing and Development, 184, 111160. 10.1016/j.mad.2019.111160 31634486

[acel13551-bib-0028] Wong, T. Y. , Solis, M. A. , Chen, Y. H. , & Huang, L. L. (2015). Molecular mechanism of extrinsic factors affecting anti‐aging of stem cells. World Journal of Stem Cells, 7(2), 512–520. 10.4252/wjsc.v7.i2.512 25815136 PMC4369508

[acel13551-bib-0029] Yoshino, J. , Baur, J. A. , & Imai, S. I. (2018). NAD(+) intermediates: The biology and therapeutic potential of NMN and NR. Cell Metabolism, 27(3), 513–528. 10.1016/j.cmet.2017.11.002 29249689 PMC5842119

[acel13551-bib-0030] Zlatanova, J. , Seebart, C. , & Tomschik, M. (2007). Nap1: taking a closer look at a juggler protein of extraordinary skills. The FASEB Journal, 21(7), 1294–1310. 10.1096/fj.06-7199rev 17317729

