# Supplementary material for: NAP1L2 drives mesenchymal stem cell senescence and suppresses osteogenic differentiation
Source: Aging Cell. 2022 Jan 15;21(2):e13551. doi: 10.1111/acel.13551 (PMC8844120; doi:10.1111/acel.13551)
Supplement: Supplementary file 1 — Supplementary Material [file ACEL-21-e13551-s001.docx]

Quantification of all the telomere dysfunction data.

Note: Data were shown as mean ± SD for at least three independent experiments.

**Figure 2 d**

**
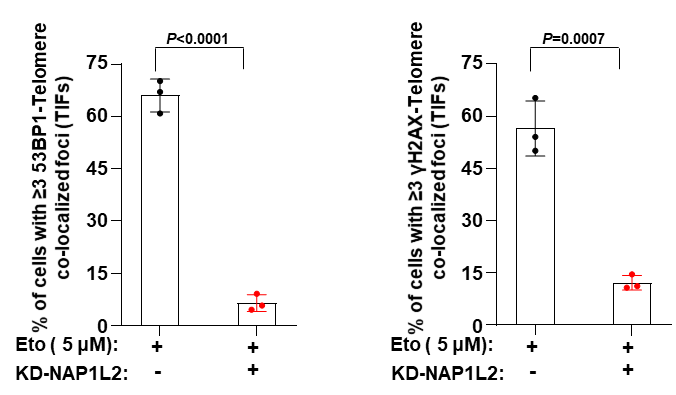
**

**Figure 2 g**


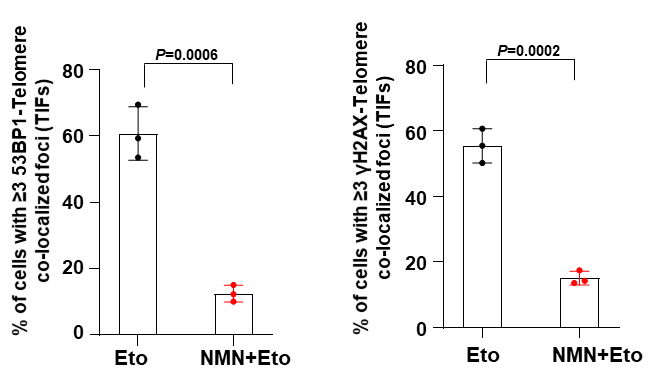


**Figure 3 b**

**
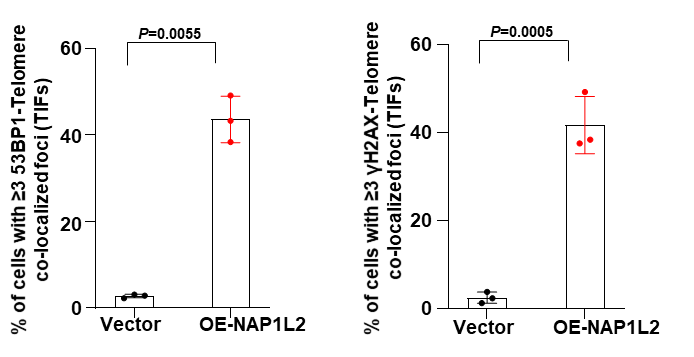
**

**Figure 4 g**

**
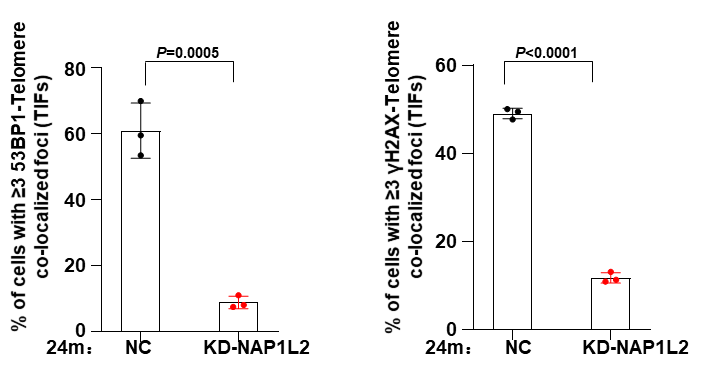
**

**Figure 6 e**

**
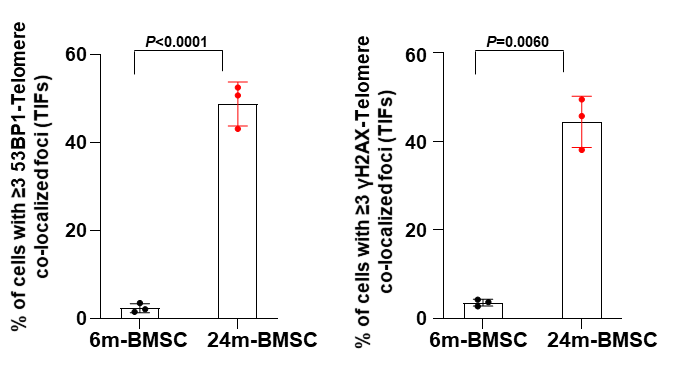
**

**Figure S2 g**

**
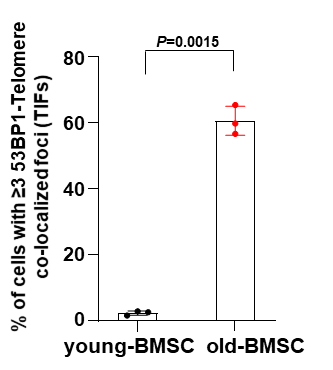
**

**Figure S2 h**

**
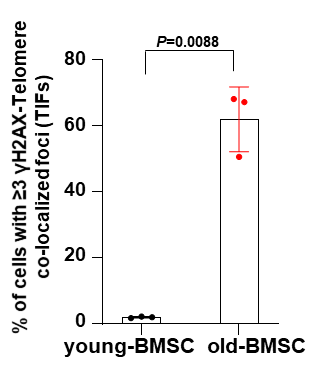
**

**Figure S5 a**


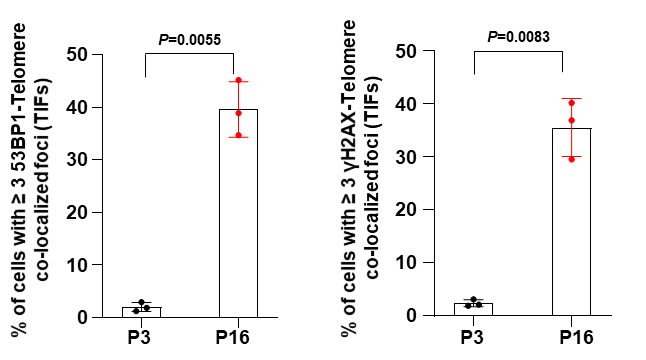


**Figure S6 b**


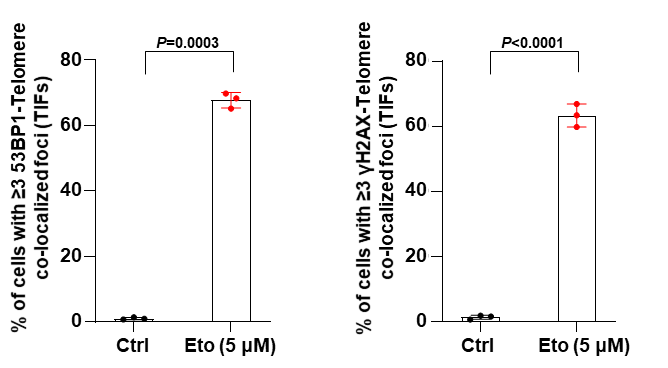


**Figure S6 g**

**
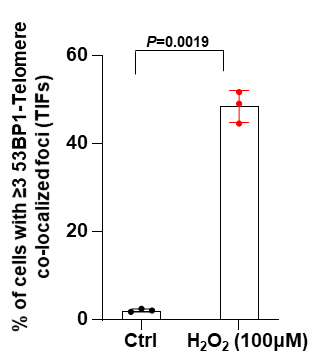
**

**Figure S6 h**

**
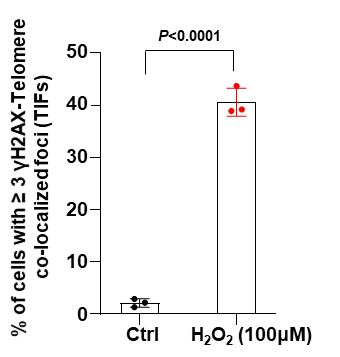
**

**Figure S8 f**

**
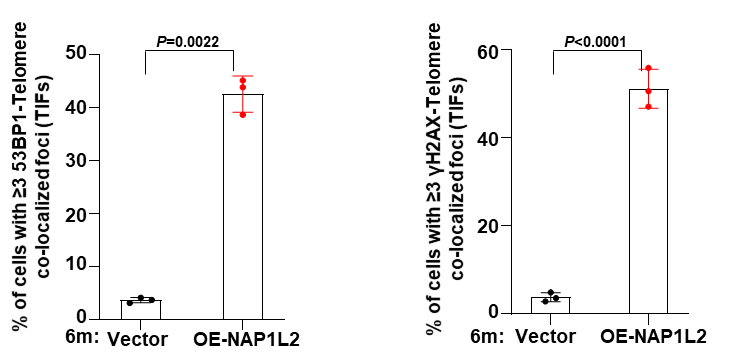
**
