# Supplementary material for: NAP1L2 drives mesenchymal stem cell senescence and suppresses osteogenic differentiation
Source: Aging Cell. 2022 Jan 15;21(2):e13551. doi: 10.1111/acel.13551 (PMC8844120; doi:10.1111/acel.13551)
Supplement: Supplementary file 2 — Fig S1‐S8 [file ACEL-21-e13551-s003.docx]

**Supplementary Figures**


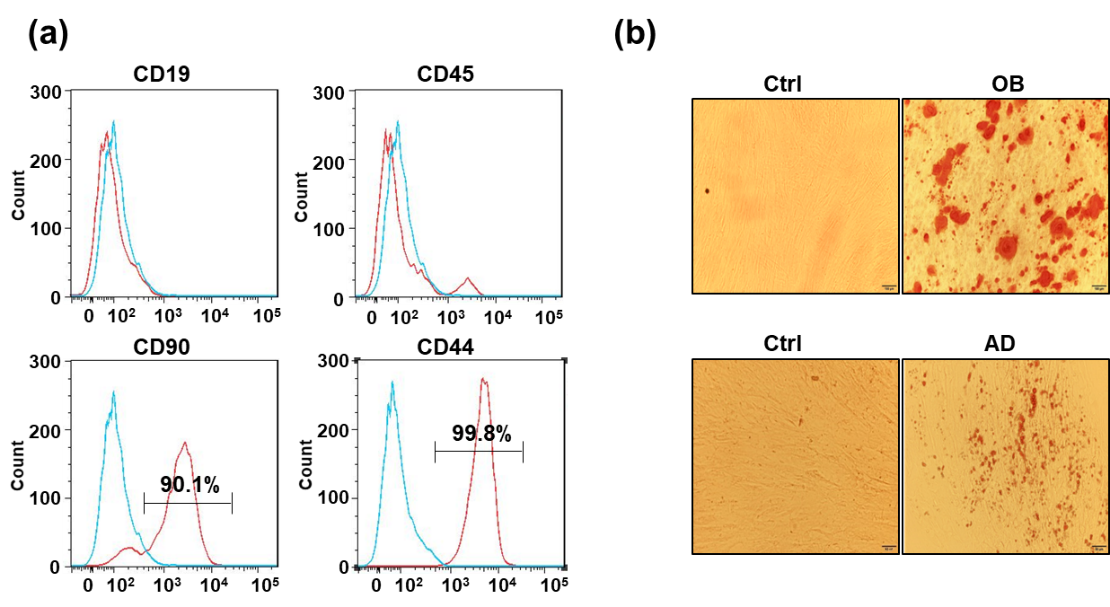


**Figure S1 Multi-lineage differentiation of BMSCs and flow cytometry analysis on biomarkers of BMSCs.**

**(a)** Flow cytometry showing the expression of surface markers CD19, CD45, CD90, and CD44 in BMSCs. **(b)** Alizarin red staining and oil red O staining showing the multidirectional differentiation ability of isolated cells. Scale bar, 50μm.


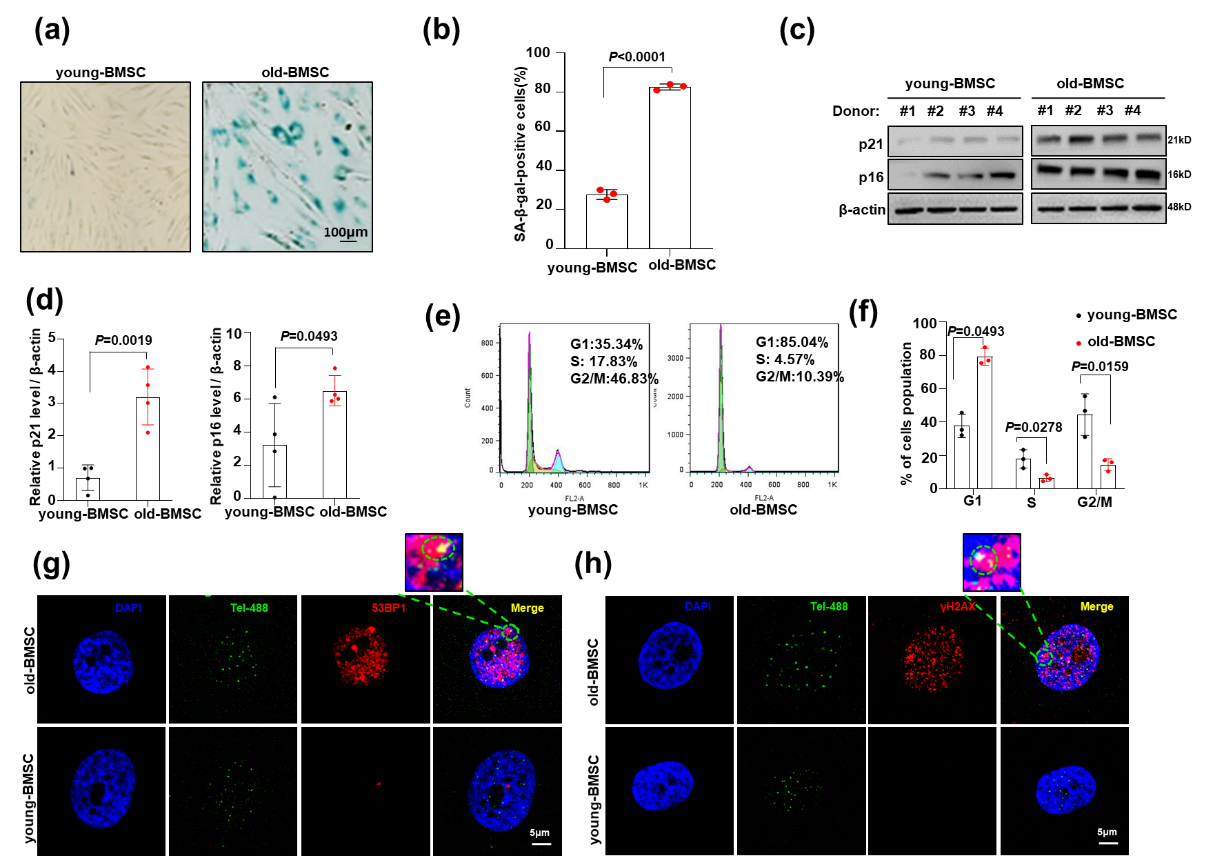


**Figure S2 BMSCs from elderly donors manifest senescent phenotypes.**

**(a)** β-galactosidase staining and **(b)**quantitative analysis in old and young BMSCs. Scale bar, 100μm (n = 3 random vision fields with 200× magnification were analyzed). **(c)** Western blot and **(b)** quantitative analysis showing protein levels of p21, p16 in old and young groups (n = 4). **(e)** Determining cell cycle stages of different age BMSCs by flow cytometry. **(f)** Quantitative analysis of **Figure S2e**. Representative images for 53BP1 **(g)** and γ-H2AX **(h)** at telomeres in old BMSCs (n = 3) and young BMSCs (n = 3). Scale bars, 5 μm.


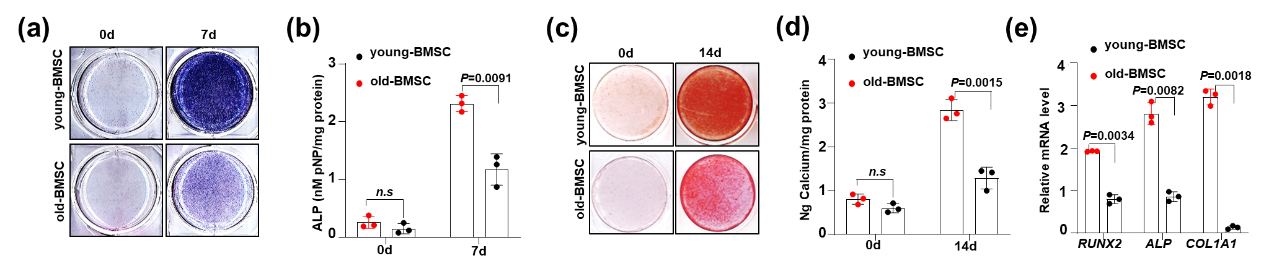


**Figure S3 BMSCs from elderly donors manifest suppressed osteogenic differentiation.**

**(a, b)** Alkaline phosphatase staining (ALP) or **(c, d)**Alizarin red staining to detect the alkaline phosphatase enzyme activity or mineralization ability of BMSCs with age (n = 3). **(e)** qPCR to show the expression levels of *RUNX2, COL1A1,* and *ALP* in the young and the old BMSCs (n = 3). *P* values of mean ± SD were determined by Student’s *t* test (n = 3).


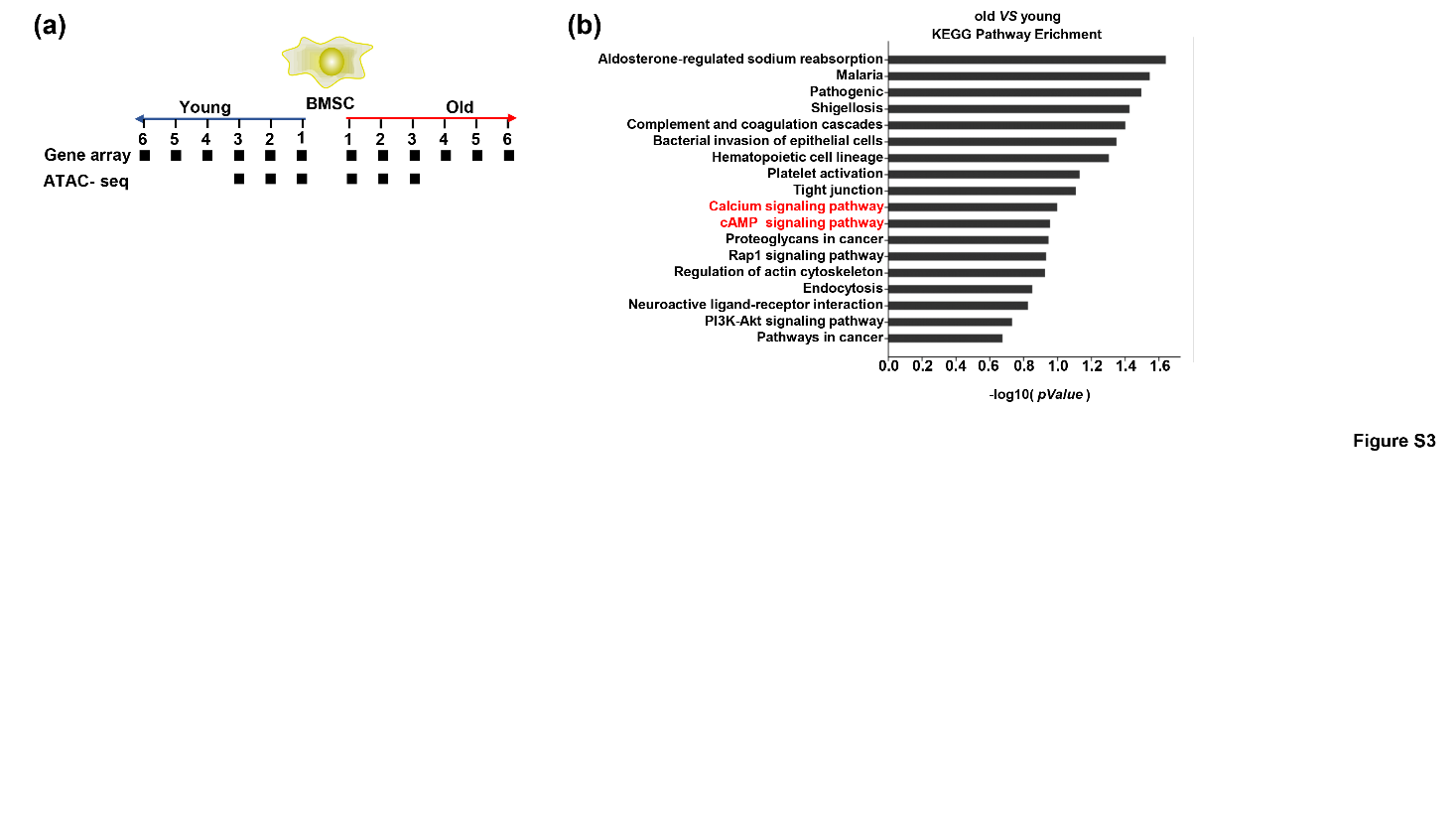


**Figure S4 Gene Array and ATAC-seq analysis of senescent BMSCs.**

**(a)** Schematic diagram illustrating the workflow of high-throughput sequencing. **(b)** KEGG Pathway analysis showing the top related pathway in old BMSCs.


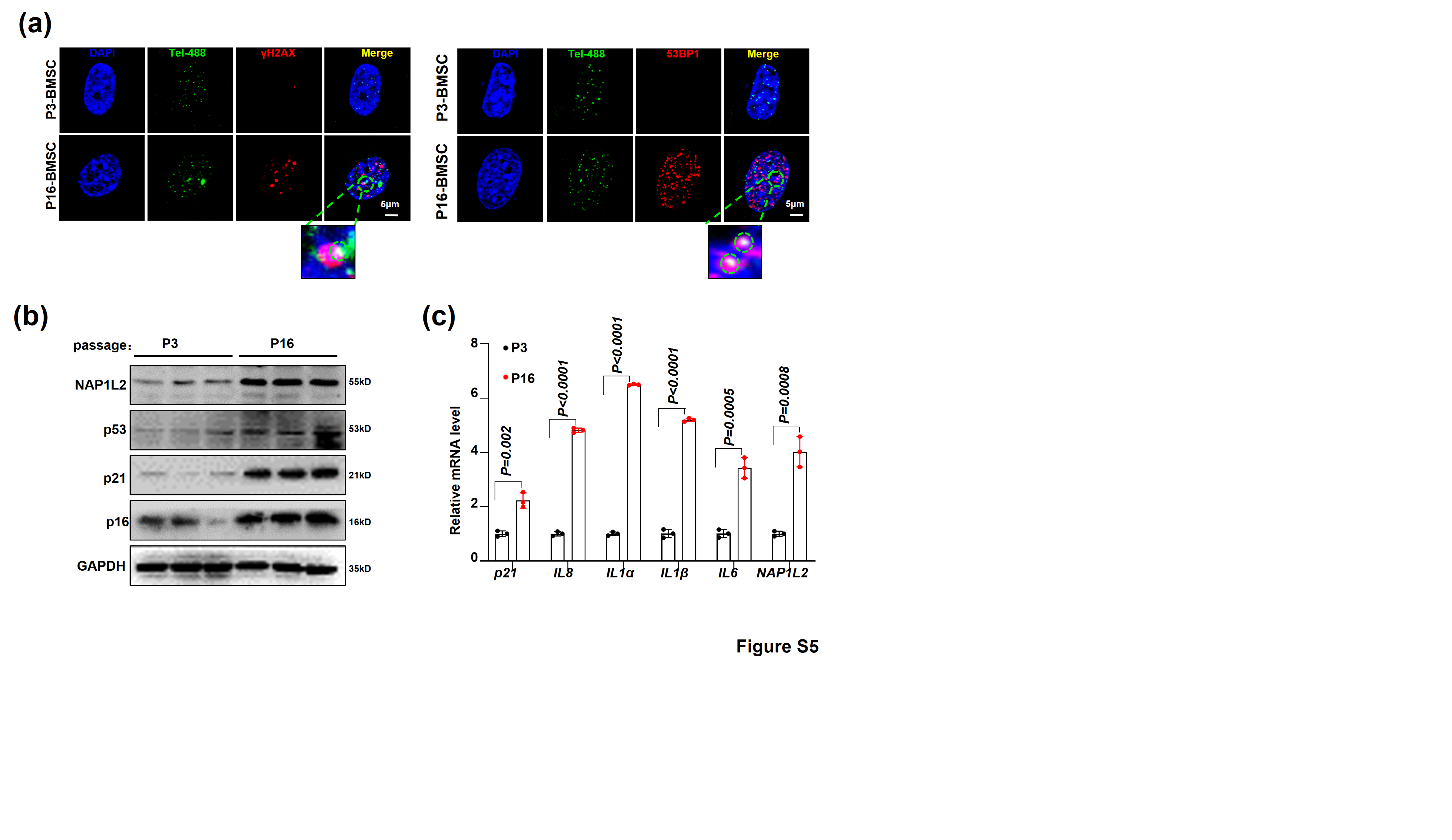


**Figure S5 Highly expressed NAP1L2 in replicative senescence BMSCs.**

**(a)** Representative images for 53BP1 and γ-H2AX at telomeres in passage 3 BMSCs (n = 3) and passage 16 BMSCs (n = 3). Scale bars, 5 μm. **(b)** Western blot showing protein levels of NAP1L2, p53, p21, and p16 in passage 3 BMSCs (n = 3) and passage 16 BMSCs (n = 3). **(c)** qPCR demonstrating the expression of *NAP1L2* and senescence-related secreted phenotype (SASP) factors, such as *IL6, IL8, IL1α, IL1β* of passage 3 BMSCs (n = 3) and passage 16 BMSCs (n = 3). *P* values of mean ± SD were determined by Student’s *t* test (n = 3).

**
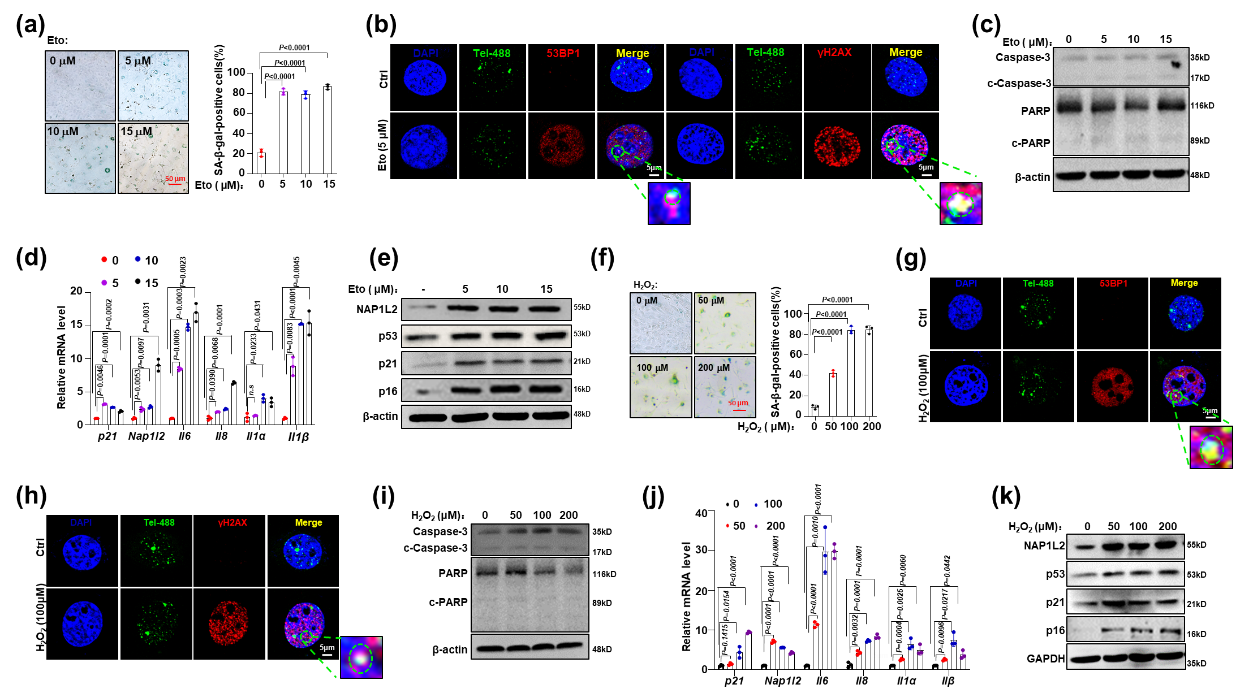
**

**Figure S6 NAP1L2 expression is elevated in chemotherapeutic agents-induced and oxidative stress-induced cellular senescence models.**

**(a)** β-galactosidase staining and quantitative analysis of C3H10T1/2 cells treated with increasing dosages etoposide (0~15 μM) for 48 hr (n = 3). Scale bar, 50μm. **(b)** Telomere damage shown by the colocalization of the telomere probe and DNA damage marker 53BP1/γ-H2AX (TIFs). Scale bars, 5 μm. Telomere probe, (CCCTAA)3-lexa488 (green); γH2AX and 53BP1, red fluorescence. **(c)** Western blot showing the protein levels of cleaved caspase-3 and cleaved PARP with increasing dosages etoposide (0~15 μM) treatment for 48 hr. **(d)** qPCR demonstrating the expression of senescence-related secreted phenotype (SASP) factors in C3H10T1/2 cells treated with increasing dosages etoposide (0~15 μM) for 48 hr (n = 3). **(e)** Western blot validating the protein levels of NAP1L2, p53, p21, and p16 with increasing dosages etoposide (0~15 μM) treatment for 48 hr. **(f)** β-galactosidase staining and quantitative analysis of C3H10T1/2 cells treated with increasing dosages H_2_O_2_ (0~200 μM) for 2 hr and maintained in 10 μM H_2_O_2_ for an additional 48 hr. Scale bar, 50μm. Telomere damage shown by the colocalization of the telomere signals and DNA damage marker 53BP1 **(g)** and γ-H2AX **(h)** in C3H10T1/2 cells treated with H_2_O_2_ or vehicle. Scale bars, 5 μm. **(i)** Western blot showing the protein levels of cleaved caspase-3 and cleaved PARP with H_2_O_2_ (0~200 μM) treatment for 2 hr and maintained in 10 μM H_2_O_2_ for an additional 48 hr. **(j)** qPCR demonstrating the expression of senescence-related secreted phenotype (SASP) factors in C3H10T1/2 cells treated with increasing dosages H_2_O_2_ (0~200 μM) for 2 hr and maintained in 10 μM H_2_O_2_ for an additional 48 hr (n = 3). **(k)** Western blot validating the protein levels of NAP1L2, p53, p21, and p16 with H_2_O_2_ (0~200 μM) treatment for 2 hr and maintained in 10 μM H_2_O_2_ for an additional 48 hr. *P* values of mean ± SD were determined by Student’s *t* test (n = 3).

**
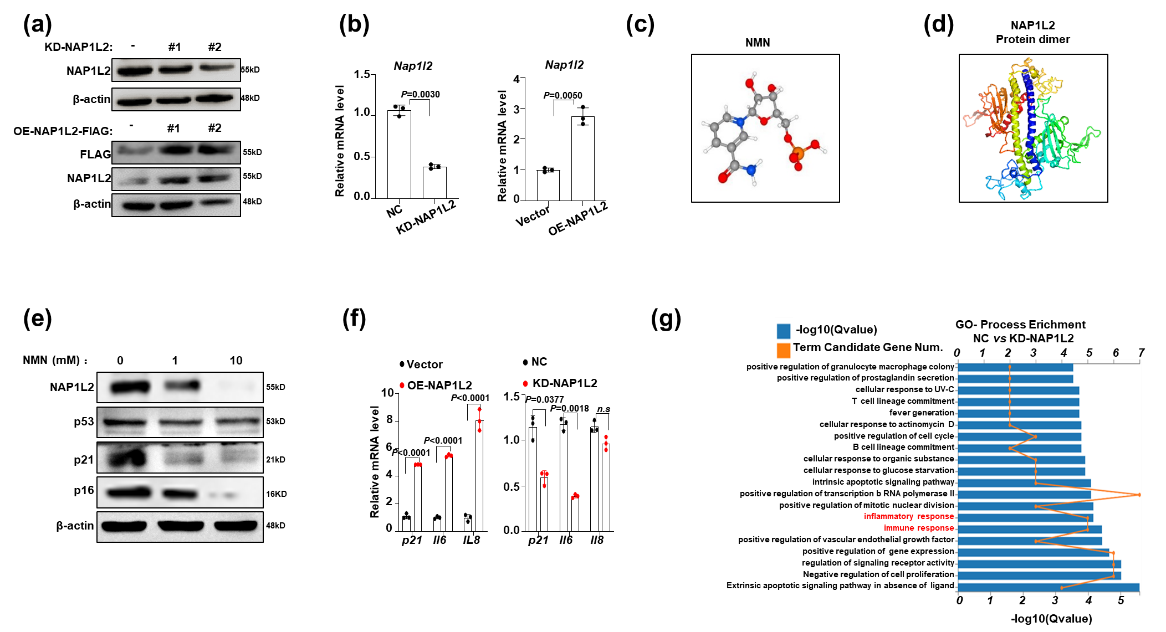
**

**Figure S7 NAP1L2 participates in the regulation of cellular senescence.**

**(a)** Western blot and **(b)** qPCR showing knocked down or overexpressed NAP1L2 in the mouse mesenchymal stem cell line C3H10T1/2 (n = 3). **(c)** The 3D structure of small molecules (NMN) was downloaded from PubChem**. (d)** Using the Swiss model server to predict NAP1L2 protein dimer structure. **(e)** Protein levels of NAP1L2, p53, p21, and p16 in C3H10T1/2 cells treated with increasing dosages NMN for 48 hr. **(f)** qPCR showing *p21* and SASP factors mRNA levels after overexpression or knockdown of NAP1L2 (n = 3). **(g)** GO analysis showing biological processes of the DEGs involved in the KD-NAP1L2 C3H10T1/2 cells. *P* values of mean ± SD were determined by Student’s *t* test (n = 3).

**
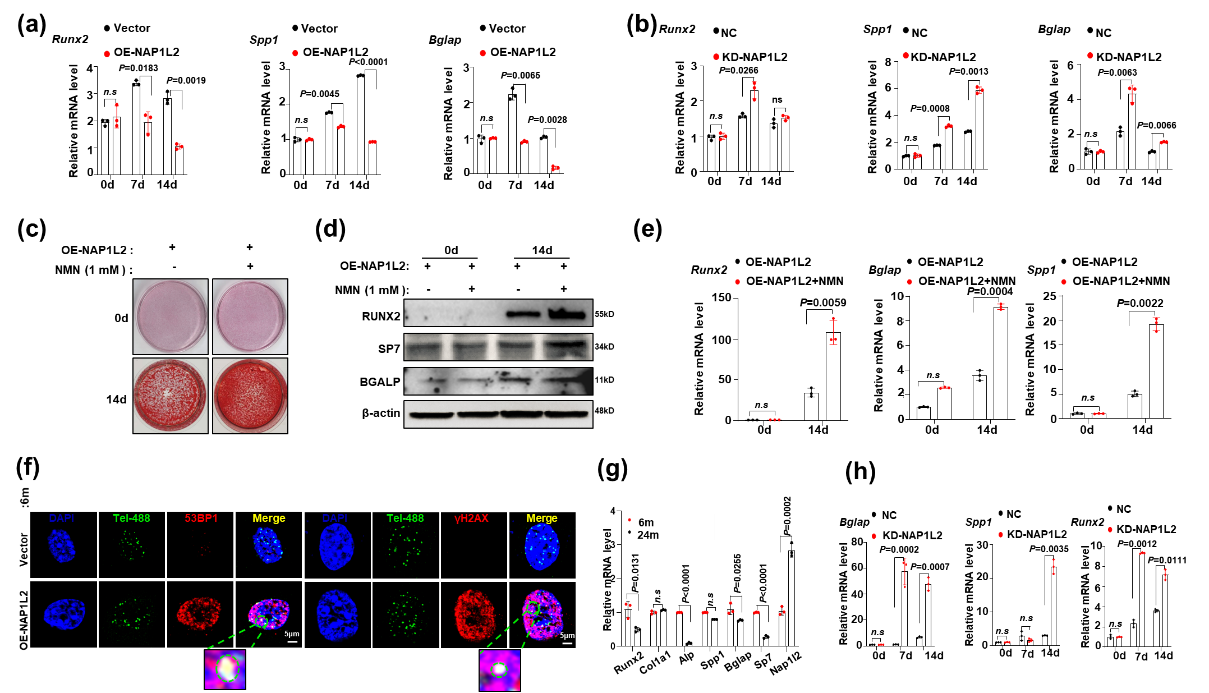
**

**Figure S8 NMN rescues the osteogenic capability of C3H10T1/2 cells overexpressing NAP1L2.**

**(a, b)** qPCR to detect the expressions of osteogenesis-related genes *Runx2*, *Spp1*, *Bglap* after NAP1L2 overexpression or knockdown under the osteogenic induction. (n = 3). *P* values of mean ± SD determined by Student’s *t* test (n = 3). **(c)** Alizarin red staining showing the osteogenic capability in overexpressed NAP1L2 C3H10T1/2 cells treated with NMN (1 mM) under osteogenesis induction**. (d, e)** Western blot and qPCR showing the expression of osteogenic genes in overexpressed NAP1L2 cells treated with NMN (1 mM) under osteogenesis induction 14days (n = 3). **(f)** Representative images for 53BP1 and γ-H2AX at telomeres after overexpression of NAP1L2 in BMSCs from young mice (n = 3). Scale bars, 5 μm. **(g)** qPCR to detect the expressions of osteogenesis-related genes and NAP1L2 in primary BMSCs of 6 months mice and 24 months mice(n = 3). **(h)** qPCR showing the expression of *Runx2*, *Spp1*, and *Bglap* in 24 months mice BMSCs after knocking down NAP1L2 under osteogenesis induction 7 days or 14 days (n = 3)*. P* values of mean ± SD were determined by Student’s *t* test.
