# Supplementary material for: NAP1L2 drives mesenchymal stem cell senescence and suppresses osteogenic differentiation
Source: Aging Cell. 2022 Jan 15;21(2):e13551. doi: 10.1111/acel.13551 (PMC8844120; doi:10.1111/acel.13551)
Supplement: Supplementary file 3 — Method S1 [file ACEL-21-e13551-s002.docx]

**Supplementary methods and materials**

**Microarray and bioinformatics analysis**

Add 1 ml Trizol (Life Technologies, USA) lysate to BMSC of different age groups, collect the lysate in 1.5 ml ribozyme-free EP tubes, and store it in -80 ° C refrigerator. After collecting six samples in each group, perform gene array sequencing. The Agilent Sure Print G3 Human Gene Expression v3 Microarray (8*60K, Design ID: 072363) (Oebiotech, China) was used in this experiment. Differentially expressed genes were then identified through fold change as well as *P* value calculated with *t*-test.

**RNA-seq**

Normal and knockdown Nap1l2 C3H10T1/2 cells were divided into the following four groups depending on whether osteogenesis induction is performed: NC, KD-Nap1l2, NC-7day, NC-Nap1l2-7day. Briefly, total RNA from treated samples was extracted using Trizol reagent (Life Technologies, USA). The construction of the RNA-seq library and the sequencing were done by Beijing Genomics Institute (Shenzhen, China). All mRNA samples were prepared for RNA-Seq analysis according to the manufacturer's protocol.

**Flow Cytometry to detect the cycle of BMSCs**

Culture three groups of cells in the same way, and the cells were digested into single cells. A total of 5×10^5^ cells were washed twice with PBS and fixed at 4°C overnight with 70% alcohol. The cells were stained with 1 μL of PI in the dark, and the cell cycle of different groups was detected by flow cytometry.

**Senescence-associated β-galactosidase assay**

Cellular senescence was assessed using the β-galactosidase staining kit following the manufacturer’s instruction (Beyotime, China). Cells were washed twice with PBS and fixed with β-galactosidase staining fixative at room temperature for 15 minutes. Then cells were stained with cell aging β-galactosidase staining solution prepared according to the instructions at 37 ° C overnight. Cells were analyzed for blue staining by randomly choosing 10 microscopic fields under a phase-contrast microscope (Olympus, Tokyo, Japan).

**Osteogenesis induction**

MSCs were cultured in a 6-well plate with complete medium. After reaching 80% confluence, the medium was changed to osteogenic differentiation medium for 7/14 days with a medium change every 3 days. The osteogenic differentiation medium was composed of high-glucose DMEM, penicillin (100 U/mL), streptomycin (100 µg/mL), dexamethasone (0.1 μmol/L), 10% FBS, ascorbic acid (50 μg/mL), and β-glycerol phosphate (10 mmol/L).

**Alkaline phosphatase assay (ALP) and quantification.**

After osteogenic differentiation, the culture medium was aspirated and the cells were fixed with 10% neutral formalin buffer for 10 minutes, washed three times with 1×PBS, and stained with alkaline phosphatase dyeing working solution (Beyotime) at room temperature in dark for 10 minutes or longer, until the color developed to the desired depth. Removed the dyeing working solution and wash it with deionized water for 1-2 times to stop the color reaction. The images were captured with a visible light microscope. To quantify the ALP activity in control and osteoblast-differentiated MSCs, we used the Alkaline Phosphatase Assay Kit (BioVision) with modified protocols. Each experiment was performed in triplicate.

**Real-time reverse transcription-polymerase chain reaction (real-time RT-PCR) analysis**

According to the manufacturer's protocol, the total cellular RNA was extracted from cells with Trizol (Life Technologies, USA) reagents and performed reverse transcription. qPCR reactions were performed by using the SYBR Green PCR kit (abm, Canada). Each sample was repeated at least three times. Expression levels were normalized relative to the expression of GAPDH. For quantification of gene expression, the ΔΔCt method was used. Primers for specific genes are listed in the Table S1.

**Western blot analysis**

Cells were harvested in RIPA buffer (50 mM Tris-Hcl, pH 7.5, 150 mM NaCl, 10 mM EDTA, 0.5% sodium deoxycholate, 1% NP-40, 1 mM sodium ovanadate, 10 μg/mL aprotinin, 1 mM phenylmethanesulfonyl fluoride, and 10 μg/mL leupeptin) with a protease inhibitor cocktail (Cell signaling technology, USA) to extract total protein, the protein concentration was measured using a BCA protein assay (ThermoFisher Scientific, USA). Histones were isolated by acid extraction. The samples were separated on 10% and 15% SDS polyacrylamide gel and transferred to nitrocellulose membranes using a semi-dry transfer apparatus. The membranes were blocked with a 5% milk solution for 2 h at room temperature and incubated with primary antibodies overnight. The following primary monoclonal antibodies were used in this study: anti-NAP1L2, anti‐FLAG, anti-H3K9ac, anti-H3K14ac, anti-H3K27me2, anti-H3K36me2, anti-H3K79me3, anti-H3K27ac, anti-H3, anti-phosphorylate p65, anti-p65, anti-RUNX2, anti-SP7, anti-BGLAP, anti-Lamin B, anti-p21, anti-p16, anti-GAPDH, anti-p53, anti-β-actin. The secondary antibody was used for 2h at room temperature. The study with three replicates was repeated at least three times. **All antibodies, venders were provided in the KEY RESOURCES TABLE.**

**Immunohistochemistry**

The femur samples were perfused with 10% neutral buffered formalin, and bone samples were decalcified in ethylene diamine tetraacetic acid for 1 month. The decalcified bone tissues were embedded in paraffin and cut into 5-µm-thick sections, deparaffinized, and rehydrated. The slices were washed three times with phosphate-buffered saline (PBS), and endogenous peroxidase was quenched following incubation with 3% hydrogen peroxide. The primary antibody used was polyclonal rabbit anti-NAP1L2 antibody and anti-LEPR antibody. The negative control slide was instead with rabbit immunoglobulin (IgG). After washing three times with PBS, slices were incubated with the secondary antibody (Maxim Ltd., China) for 30 min, followed by 3, 3′-diaminobenzidine (DAB) development (Thermo Scientific, USA). Finally, nuclei were localized by heamtoxylin staining for 1-2 min before mounting and capture.

**Immunofluorescence staining (IF)**

For IF assays, C3H10T1/2 cells were fixed in 4% formaldehyde, then samples were permeabilized and blocked. After incubation with anti-p65 antibody (1:500) or anti-FLAG antibody (1:200) overnight at 4°C, samples were incubated with Alexa Fluor^TM^ 555-conjugated secondary antibody or Alexa Fluor^TM^ 488-conjugated secondary antibody (1:2000) for 60 min at room temperature and nuclei were counterstained with DAPI. Signal detection was carried out by fluorescence imaging performed using an Olympus FV1000 IX81-SIM Confocal Microscope (Olympus, Tokyo, Japan).

**Simulation of binding of NAP1L2 and anti-senility drugs (NMN)**

Auto-Dock 4.0 was used to dock small molecules (anti senility Drugs (NMN)) into the NAP1L2 protein. The original NAP1L2 protein structure was predicted using the Swiss model server. The structure of small molecules (NMN) was download from PubChem. The information on active sites and substrate binding sites of NAP1L2 was predicted from the COACH website. Before the docking simulation, the small molecule (NMN) was placed into the substrate-binding site of NAP1L2 as the start point of docking. The parameters for docking were set as follows: the Lamarckian genetic algorithm (LGA) runs were set at 100, and the maximum number of energy evaluations was set at 2.5 million. The simulation box was fixed at the center of the substrate, and the box size was set at 80 ÅX80 ÅX60 Å in all three dimensions. The small molecule conformation with the highest binding energy is considered to be the ideal binding conformation. The final data was visualized by Chimera software.

**Biomechanical testing**

The same load frame was used for all mechanical testing (Instron 8511, High Wycombe, UK). The mechanical tests were conducted at room temperature. Both the old and young mice femurs were was subjected to three-point bending test. The femurs were mounted in the AP-plane with the posterior surface of the bone resting on the two lower supports. The center–center distance between the lower supports was 10 mm. Thereafter, the bones were tested until failure at a constant speed of 1.0 mm/s whilst time. Based on the losd-displacement curve, the maximum load was determined and the elastic modulus were calculated.

**Supplementary Tables.**

**Tables S1 Primers used in this study.**

| **Primers sequence** | | |
| --- | --- | --- |
| HumanGAPDH-F | | TTGCCCTCAACGACCACTTT |
| HumanGAPDH-R | | TGGTCCAGGGGTCTTACTCC |
| Human-Runx2-F | | TAGGCGCATTTCAGGTGCTT |
| Human-Runx2-R | | GGTGTGGTAGTGAGTGGTGG |
| Human-ALP-F | | TTTATAAGGCGGCGGGGGT |
| Human-ALP-R | | TTAACTGATGTTCCAATCCTGCG |
| Human-Col1A1-F | | TGTTCAGCTTTGTGGACCTC |
| Human-Col1A1-R | | GGTGATTGGTGGGATGTCTT |
| Human-NAP1L2-F | | TCAGTCCGGTTCTCAAAGCC |
| Human-NAP1L2-R | | GTGGCTACCACAGAGATCGG |
| Human-p21-F | | GGTGGCAGTAGAGGCTATGG |
| Human-p21-R | | ATTCAGCATTGTGGGAGGAG |
| Human-p16-F | | AGCAGTCCGACTCTCCAAAA |
| Human-p16-R | | GGGTGTTTGGTGTCATAGGG |
| Human -Il6-F | | CCTTCTCCACAAACATGTAACAAGA |
| Human -Il6-R | | ACCAGGCAAGTCTCCTCATTG |
| Human -Il8-F | | AGTTTTTGAAGAGGGCTGAGA |
| Human -Il8-R | | TGCTTGAAGTTTCACTGGCATC |
| Human -Il1β-F | | AGCCATGGCAGAAGTACCTG |
| Human -Il1β-R | | CCTGGAAGGAGCACTTCATCT |
| Human -Il1α-F | | GCGTTTGAGTCAGCAAAGAAGT |
| Human -Il1α-R | | CATGGAGTGGGCCATAGCTT |
| Mus-Nap1l2-F | CGGGCTTAGCCACCATAAGT | |
| Mus-Nap1l2-R | TTCACTGCGGTCCTGACTTT | |
| Mus-Runx2-F | TCGGAGAGGTACCAGATGGG | |
| Mus-Runx2-R | TGAAACTCTTGCCTCGTCCG | |
| Mus-SPP1-F | AAGCATCCTTGCTTGGGTTTG | |
| Mus-SPP1-R | TGGTCGTAGTTAGTCCCTCAG | |
| Mus-Bglap-F | GAACAGACAAGTCCCACACAGC | |
| Mus-Bglap-R | TCAGCAGAGTGAGCAGAAAGAT | |
| Mus-Sp7-F | | GTCCTCTCTGCTTGAGGAAGAA |
| Mus-Sp7-R | | GGGCTGAAAGGTCAGCGTAT |
| Mus-p21-F | | ATCCAGACATTCAGAGCCACAG |
| Mus-p21-R | | AGACAACGGCACACTTTGCT |
| Mus-Il6-F | | CCTCTCTGCAAGAGACTTCCAT |
| Mus-Il6-R | | ACAGGTCTGTTGGGAGTGGT |
| Mus-Il8-F | | AGGAAGTGATAGCAGTCCCAA |
| Mus-Il8-R | | CAGAAGCTTCATTGCCGGTG |
| Mus-Il1β-F | | TGCCACCTTTTGACAGTGATG |
| Mus-Il1β-R | | AAGGTCCACGGGAAAGACAC |
| Mus-Il1α-F | | GCTTGAGTCGGCAAAGAAATC |
| Mus-Il1α-R | | CTGATACTGTCACCCGGCTC |
| Mus-Runx2-ChIP-F | | AGGTGGAAGACCCGTGAAGA |
| Mus-Runx2-ChIP-R | | TCTATGAAGGCATTTCCTGG |
| Mus-Sp7-ChIP-F | | AGAGAGAGGCACTGCTTAGG |
| Mus-Sp7-ChIP-R | | CCAGGCTATAATCCTCTTGG |
| Mus-Bglap-ChIP-F | | GGCTTAGGAGACAGGGTGATG |
| Mus-Bglap-ChIP-R | | TGCAGAACAGACAAGTCCCA |

**KEY RESOURCES TABLE**

| **REAGENT or RESOURCE** | **SOURCE** | **IDENTIFIER** |
| --- | --- | --- |
| **Antibodies** | | |
| Anti-NAP1L2 rabbit | ABclonal | Cat#A12087 RRID:AB_2758986 |
| Anti-H3K9ac rabbit | ABclonal | Cat#A7255 RRID:AB_2737400 |
| Anti-BGLAP rabbit | ABclonal | Cat#A18241  RRID:AB_2862017 |
| Anti-β-actin rabbit | Abclonal | Cat#AC006  RRID:AB_2768236 |
| Anti-p16 rabbit | Abclonal | Cat#A4794s  RRID:AB_2863349 |
| Anti-H3K14ac rabbit | Abcam | Cat#ab52946 RRID:AB_880442 |
| Anti-p65 rabbit | Abcam | Cat#ab16502 RRID:AB_443394 |
| Anti-p65 (phospho S536) rabbit | Abcam | Cat#ab86299 RRID:AB_1925243 |
| Anti-Histone H3 antibody - Nuclear Loading Control and ChIP Grade | Abcam | Cat#ab1791  RRID:AB_302613 |
| Anti-SP7 mouse | Abcam | Cat#ab57335 RRID:AB_944561 |
| Anti-53BP1 rabbit | Abcam | Cat# ab175933  RRID: AB_2890610 |
| Anti-RUNX2 rabbit | Cell signaling technology | Cat#12556s  RRID:AB_2732805 |
| Anti- γ-H2AX rabbit | Cell signaling technology | Cat#2577  RRID: AB_2118010 |
| Anti-Caspase-3 rabbit | Cell signaling technology | Cat#9662s  RRID: AB_331439 |
| Anti-PARP rabbit | Cell signaling technology | Cat#9542s  RRID:AB 2160739 |
| Anti-H3K27me2 rabbit | Cell signaling technology | Cat#9728T  RRID:AB_1281338 |
| Anti-H3K36me2 rabbit | Cell signaling technology | Cat#2901s  RRID:AB_1030983 |
| Anti-H3K79me3 rabbit | Cell signaling technology | Cat#74073  RRID:AB_2799849 |
| Anti-H3K27ac rabbit | Cell signaling technology | Cat#8173  RRID:AB_10949503 |
| Anti-p53 mouse | Cell signaling technology | Cat#2524s  RRID:AB_331743 |
| Anti-Lamin B goat | SANTA CRUZ BIOTECHNOLOGY | Cat#sc6216  RRID:AB_648156 |
| Anti-p21 mouse | SANTA CRUZ BIOTECHNOLOGY | Cat#sc397  RRID:AB_632126 |
| Anti-GAPDH mouse | ImmunoWay | Cat#YM3029 |
| Anti-NAP1L2 rabbit | Novus Biologicals | Cat#NBP1-57024  RRID:AB_11008915 |
| Goat Anti-Rabbit IgG-HRP | Sigma-Aldrich | Cat#A0545  RRID:AB_257896 |
| ANTI-FLAG® M2-Peroxidase | Sigma-Aldrich | Cat#A8592  RRID:AB_2769604 |
| Rabbit Anti Mouse IgG-HRP | Sigma-Aldrich | Cat#A9044-2ML  RRID:AB_258431 |
| Anti-rabbit IgG | Proteintech | Cat#30000-0-AP  RRID:AB_2819035 |
| Anti-mouse IgG | Proteintech | Cat#B900620  RRID:AB_2883054 |
| Alexa Fluor^TM^ 555-donkey anti-rabbit -IgG(H+L) | Invitrogen | Cat#A31572  RRID: AB_162543 |
| Alexa Fluor^TM^ 488-goat anti-mouse -IgG(H+L) | Invitrogen | Cat#A11001  RRID: AB_2534069 |
| **Chemicals, peptides, and recombinant proteins** | | |
| FLAG Peptide | Sigma-Aldrich | Cat#F3290 |
| PEG 8000 30% solution | Sigma-Aldrich | Cat#86686 |
| Etoposide | SelleckChem | Cat#S1225 |
| Puromycin 2HCL | SelleckChem | Cat#S7417 |
| β-Nicotinamide Mononucleotide | SelleckChem | Cat#S5259 |
| RNase A, DNase and protease-free | Thermo Fisher | Cat#EN0531 |
| Proteinase K Solution, ChIP grade | Thermo Fisher | Cat#26160 |
| Benzonase Nuclease | Sigma-Aldrich | Cat#E1014-25KU |
| XbaI | NewEngland Biolabs | Cat#R0145S |
| KpnI-HF | NewEngland Biolabs | Cat#R3142S |
| CutSmart Buffer | NewEngland Biolabs | Cat#137204S |
| NEBuffer1 | NewEngland Biolabs | Cat#B7001S |
| NEBuffer2 | NewEngland Biolabs | Cat#B7002S |
| NEBuffer3 | NewEngland Biolabs | Cat#B7003O |
| NEBuffer4 | NewEngland Biolabs | Cat#B7004S |
| T4 DNA Ligase | NewEngland Biolabs | Cat#M0202S |
| 10×Buffer for T4 DNA ligase | NewEngland Biolabs | Cat#B0202S |
| Multiscribe Reverse Transcriptase | ABI | Cat#4308228 |
| dNTP mix | ABI | Cat#362275 |
| PNA—TelC-Alexa488 | PANAGENE | Cat#NO.F1004 |
| **Critical commercial assays** | | |
| EvaGreen 2X qPCR MasterMix | ABI | Cat#MasterMix-LR |
| 5×All-In-One RT MasterMix | abm | Cat#G490 |
| Pierce BCA Protein Assay Kit | Thermo SCIENTIFIC | Cat#23225 |
| AxyPrep DNA Extraction Kit | AXYGEN | Cat#295 AP-GX-250G |
| AxyPrep Plasmid Miniprep Kit | AXYGEN | Cat#183 AP-MN-P-250G |
| Plasmid Maxi Kit(25) | QIAGEN | Cat#12163 |
| EnVision G12 Doublestain System,Rabbit/Mouse(DAB+/Permanent Red) | Dako | Cat#K5361 |
| SuperSignal West Dura Extended Duration Substrate | ThermoFisher | Cat#34580 |
| 9002 SimpleCHIP® Kit | Cell Signaling technology | Cat#22188S |
| Simple CHIP® Kits-20C-Reagents | Cell Signaling technology | Cat#45061S |
| ChIP-grade Protein A/G Magnetic Beads | Thermo SCIENTIFIC | Cat#26162 |
| ANTI-FLAG M2 Affinity Gel | Sigma-Aldrich | Cat#A2220 |
| CellTiter 96 Aqueous One Solution | Promega | Cat#G358B |
| NuPAGE 4-12% Bis-Tris Gel | Invitrogen | Cat#NP0335BOX |
| Phosphatase Inhibitor Cocktail(100×) | Cell Signaling technology | Cat#5871S |
| Pierce® Protein G Plus Agarose | Thermo Scientific | Cat#22852 |
| Ficoll-Paque PLUS endotoxin tested | GE Healthcare | Cat#17-1440-02 |
| TRIzol Reagent | Ambion, Life Science | Cat#15596018 |
| Opti-MEM®I(1×) Reduced Serum | Gibco, Life Technologies | Cat#31985-070 |
| Opti-protein XL Marker | ABM | Cat#G266 |
| PageRuler Prestained protein Ladder | ThermoFisher Scientific | Cat#26616 |
| 1Kb Ladder DNA Marker | Biomed | Cat#MD114 |
| 1Kb DNA Ladder | TIANCEN | Cat#MD111 |
| 100bp DNA Ladder | TRANS | Cat#BM301 |
| BM15000 DNA Marker | Biomed | Cat#MD106 |
| 1Kb Plus DNA Ladder | Solarbio | Cat#M1500 |
| PEI-Transferrinfection Kit | ThermoFisher Scientific | Cat#BMS1003 |
| β -galactosidase staining kit | Beyotime | Cat#C0602 |
| Alkaline phosphatase assay | Beyotime | Cat#C3206 |
| **Deposited data** | | |
| RNA-seq / ChIP-seq data | This study | GSE166244. |
| **Experimental models: cell lines** | | |
| 293T lines | Laboratory of Zhiqiang Liu | N/A |
| C3H10T1/2 lines | Laboratory of Zhiqiang Liu | N/A |
| **Experimental models: organisms/strains** | | |
| Mouse: C57BL/6J | Laboratory of Zhiqiang Liu | N/A |
| **Recombinant DNA** | | |
| hU6-MCS-Ubiquitin-EGFP-IRES-puromycin vector | Shanghai genechem | PIEE248068084 |
| mNap1l2-shRNA1 | Shanghai genechem | PIEE248068084 |
| mNap1l2-shRNA2 | Shanghai genechem | PIEE248068084 |
| mNap1l2-shRNA3 | Shanghai genechem | PIEE248068084 |
| pITA insert | Laboratory of Yupeng Chen | N/A |
| PSPAX_2_ | Laboratory of Xudong Wu | N/A |
| PMD_2_G | Laboratory of Xudong Wu | N/A |
| pITA insert-mNap1l2-FLAG | Laboratory of Zhiqiang Liu | N/A |
| pCDH-FLAG-Sirt1 | Laboratory of Zhiqiang Liu | N/A |
| pcDNA6-V5-mSirt1 | Laboratory of Zhiqiang Liu | N/A |
| **Software and algorithms** | | |
| GraphPad Prism 8 | Graphpad Software | RRID: SCR_002798 |
| FlowJo | BD | RRID: SCR_008520 |
| AutoDock 4.0 | Autodock Software | RRID: SCR_012746 |
| Swiss model server | Web Service | RRID: SCR_018123 |
| COACH for protein-ligand binding site prediction | Web Service | https://zhanglab.ccmb.med.umich.edu/COACH  RRID: SCR_014627 |
| Chimera 1.13.1 | UCSF Chimera software | RRID: SCR_004097 |
